# Imaging the in vivo growth patterns of bacteria in human gut Microbiota

**DOI:** 10.1080/19490976.2021.1960134

**Published:** 2021-08-24

**Authors:** Liyuan Lin, Jia Song, Jian Li, Xiaolei Zuo, Hong Wei, Chaoyong Yang, Wei Wang

**Affiliations:** a Institute of Molecular Medicine, Renji Hospital, Shanghai Jiao Tong University School of Medicine, Shanghai, China; b Institute of Immunology, PLA, Third Military Medical University, Chongqing, China; c Central Laboratory, Clinical Medicine Scientific and Technical Innovation Park, Shanghai Tenth People’s Hospital, Tongji University, Shanghai, China; d State Key Laboratory of Agricultural Microbiology, College of Animal Sciences and Technology, Huazhong Agricultural University, Wuhan, P. R. China; e The MOE Key Laboratory of Spectrochemical Analysis and Instrumentation, Key Laboratory for Chemical Biology of Fujian Province State Key Laboratory of Physical Chemistry of Solid Surfaces, Department of Chemical Biology, College of Chemistry and Chemical Engineering, Xiamen University, Xiamen, China

**Keywords:** Human gut microbiota, metabolic labeling, D-amino acids, peptidoglycan, STAMP, FISH, bacterial division patterns, fluorescence imaging, Microbiome Atlas

## Abstract

How to study the unculturable bacteria in the laboratory is one of the major challenges in human gut microbiota research. The resulting lack of microbiology knowledge of this “dark matter” greatly hinders further understanding of our gut microbiota. Here, to characterize the *in vivo* growth and division of human gut bacteria, we report the integrative use of STAMP (sequential tagging with D-amino acid–based metabolic probes) and fluorescence *in situ* hybridization (FISH) in a human microbiota-associated mouse model. After stable colonization of the human fecal microbiotas in germ-free mice, two fluorescent D-amino acid probes were sequentially administered by gavage, and the dually labeled peptidoglycan of the bacteria provided a chronological recording of their cell wall syntheses. Following taxonomic identification with FISH staining, the growth patterns of 32 species, including 5 currently unculturables, were identified. Surprisingly, we found that many bacterial species in the human microbiota were significantly shorter than those in the mouse gut microbiota. An imaging database for gut bacteria – Microbiome Atlas was built for summarizing STAMP imaging of bacteria from different microbiotas, which can be contributed by the microbiota research community worldwide. This integrative imaging strategy and the database will promote our understanding of the bacterial cytology in gut microbiotas and facilitate communications among cellular microbiologists.

## Introduction

Bacteria in the human gut microbiota possess tremendous biodiversity, but many still cannot be cultured separately *in vitro*, preventing them from any laboratory studies. Other than the genomic information, little is known about the basic microbiology of these bacteria, many of which have important physiological or pathological functions in the gut.^1,2^ Moreover, for the culturable bacteria, the knowledge of microbial cytology obtained from *in vitro* measurements may not be representative of what truly happens *in vivo*. A method that can characterize the microbial properties of this gut “dark matter” without the need of *in vitro* culture is, therefore, highly desirable.^3,4^ Recently, we reported a strategy that could reveal the indigenous growth and division patterns of different bacterial genus or species in the gut microbiota of mice and rats.^5,6^ This integrative method combined the use of *in vivo* sequential tagging with fluorescent D-amino acid-based probes (STAMP) and fluorescent *in situ* hybridization (FISH).^5^ Fluorescent D-amino acid (FDAA) probes can metabolically label peptidoglycan (PGN) by the functioning of bacterial D,D and L,D-transpeptidases, which play pivotal roles in their PGN constructions.^7,8^ The sequential labeling with gavaged FDAAs containing different fluorophores chronologically recorded the morphogenesis of most bacteria in the gut, including those that had not been cultured individually *in vitro*. The subsequent use of FISH staining taxonomically identified the STAMP-labeled bacteria and revealed the growing status of different gut bacteria *in situ*.

It’s well recognized that the bacterial compositions are very different between the human and mouse gut microbiotas.^9,10^ Therefore, it’s of great value to investigate the growth and division patterns of human gut bacteria through this integrative strategy. Due to the absorption of FDAA probes into the host circulation,^11^ the employment of this protocol to directly probe human gut microbiota, however, is potentially hazardous and thus impractical. To solve this problem, here we propose the use of the STAMP+FISH strategy in a human microbiota-associated (HMA) mouse model. After successfully constructing the HMA mouse models by colonizing human fecal microbiota in germ-free mice, two FDAAs were sequentially given to mice by gavage. Following taxonomic identification with FISH staining, we determined the *in vivo* growth and division patterns of 32 bacterial species based on their FDAA labeling signals. It provides a unique opportunity to explore the microbial cytology in the human gut microbiota, which can be compositionally and phenotypically different from the mouse native microbiota.

## Results

### STAMP labeling of the HMA mouse gut microbiota

**Figure 1.**
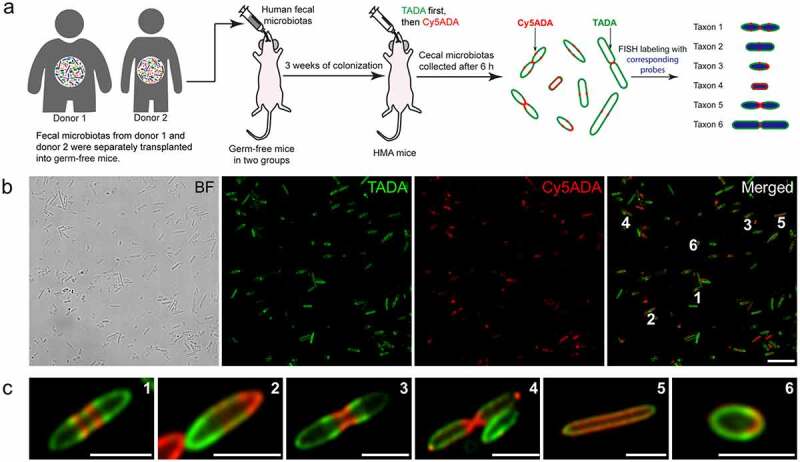
Schematic illustration of the labeling strategy used in this study, and the two-color fluorescence imaging of the STAMP-labeled human-derived microbiota. (a) The fecal microbiotas from human feces were transplanted to two groups of GF mice separately and the HMA mice then received sequential gavage of TADA and Cy5ADA. The cecal microbiotas of HMA mice was collected and imaged, with their taxonomic identifications determined by separate FISH staining. (b) Two-color fluorescence imaging of the human-derived bacteria sequentially labeled by TADA (green) and Cy5ADA (red). BF, bright field. Scale bar, 10 μm. Representative images from at least three independent experiments are shown. (c) Zoomed views of the indicated bacteria from the merged image above. The green and red signals of the two FDAAs revealed the distinct growth patterns of the bacteria. Scale bars, 2 μm

We collected fecal microbiotas from two healthy volunteers (donor 1 and 2), and separately transplanted them to two groups of germ-free BALB/c mice (five in each group). After three weeks of colonization, STAMP labeling was performed using two FDAA probes, TAMRA-amino-D-alanine (TADA) and Cy5-amino-D-alanine (Cy5ADA), functionalized with TAMRA (tetramethylrhodamine) or Cy5 (Cyanine 5) on the side chains, in gavages at an interval of 3 h to label the human-derived microbiota (scheme shown in Figure 1(a), the FDAA-labeling could last for >10 h according to our previous report^7^).

For each group of HMA mice, their cecal microbiotas were collected and combined for further analyses. Under fluorescence microscope, great morphological diversities were observed in the microbiotas (Figure 1(b)), and high percentages of the bacteria (70.87% and 67.30% for the two groups, respectively, Figure S1) were labeled with FDAA probes, indicative of active metabolism of the human gut bacteria in their new hosts. Different distributions of the two-color fluorescence among various bacteria provided a chronological recording of the PGN synthesis in each bacterium, and stronger labeling of the second FDAA probe (Cy5ADA, shown in red) represented sites with more active PGN constructions.^5^ Most bacteria exhibited zonal growth, where the addition of new PGN was concentrated to a specific area, such as elongating near the division plane (Figure 1(c), No. 1), growing at one pole of the cell (Figure 1(c), No. 2), or forming septum at the midcell (Figure 1(c), No. 3, 4). Some rod-shaped bacteria elongated through dispersed lateral growth (Figure 1(c), No. 5). Besides these long rod/spindle-shaped bacteria, the division patterns of small rod/round-shaped bacteria could also be readily observed (Figure 1(c), No. 6).

To facilitate the following FISH probe selection and design, we performed metagenomic sequencing of the microbiotas. Fifty-two species with relative high abundance (>0.1%) were identified from the two groups in total (Table S1). Not surprisingly, the abundances of the microbiotas on the species level were very different between the two groups, with some species only existing in one group. We also performed 16S rRNA gene sequencing to compare the colonized microbiotas in HMA mice and the donor microbiotas from human feces (Figure S2), and found that ~70% genera detected in the human fecal samples were also found in the recipient mice (Table S2).

### Identify the bacterial growth patterns on the species level

We selected 42 species from the two microbiotas and labeled them with corresponding FISH probes separately to visually identify the bacteria. The FISH probes used were either previously used in literatures or designed *de novo* using an algorithm previously reported.^5,12^ In this study, we further optimized the algorithm to prevent the formation of secondary DNA structures and improve the hybridization success rates of the designed sequences. The specificities of the designed FISH probes targeting each species were carefully verified from multiple aspects: 1) consistent labeling patterns and morphogenesis of the bacteria stained by the same probe (Figure S3); 2) if possible, agreeing results between the labeling ratio of the FISH-stained bacteria by flow cytometry and the corresponding relative abundance determined by metagenomic sequencing (Figure S4); 3) when some species only existed in either the donor 1 or the donor 2 group, the two groups of human-derived microbiotas were used as the control group for each other when testing FISH probes of these species. In all cases, the group not having a particular species according to DNA sequencing indeed lacked the corresponding FISH signals (Table S3).

**Figure 2.**
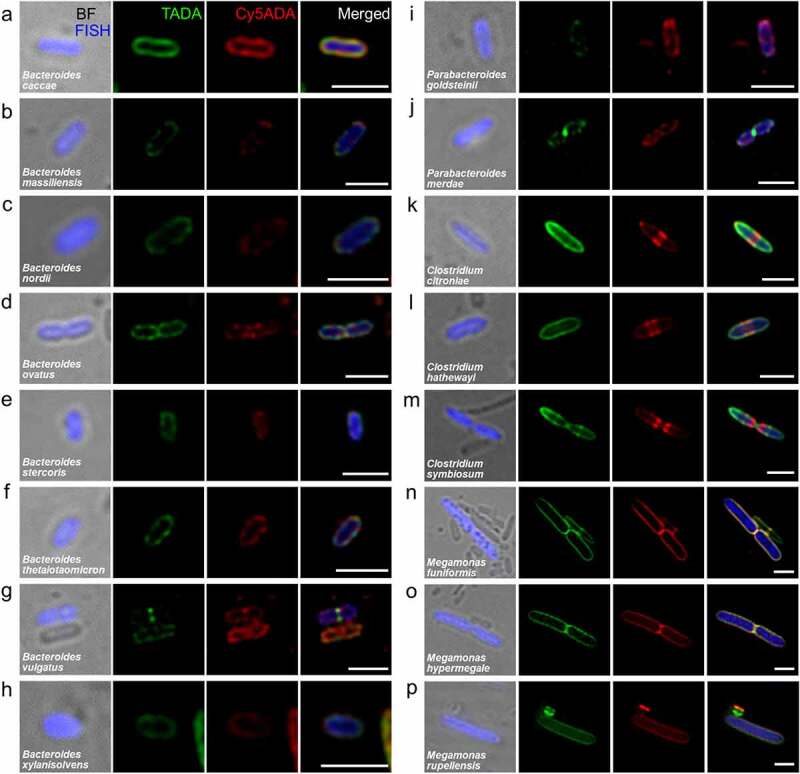
Confocal fluorescence imaging of 16 STAMP-labeled and FISH-stained Gram-negative species from the two human-derived microbiotas. The cecal microbiotas of HMA mice received sequential labeling of TADA (green) and Cy5ADA (red) were stained by different FISH probes (blue) targeting corresponding species. Representative images of FDAA-labeled bacterial species belonging to the families Bacteroidaceae (a-h), Porphyromonadaceae (i, j), Clostridiaceae (k-m) and Veillonellaceae (n-p) are shown. Scale bars, 2 μm. Representative photographs of bacteria, which showed consistent labeling pattern in each species from at least three independent FISH experiments, are shown

**Figure 3.**
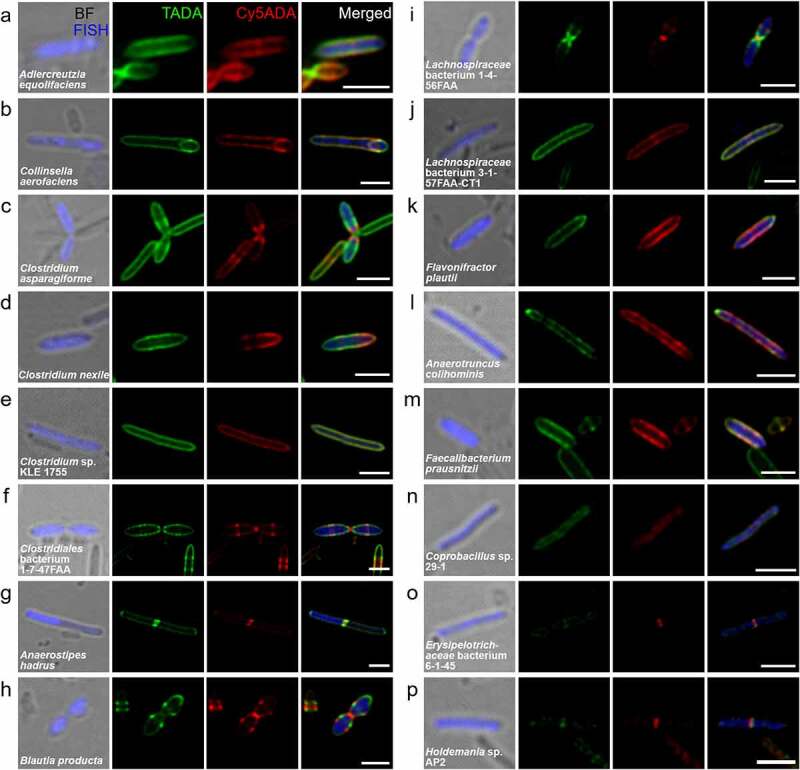
Confocal fluorescence imaging of 16 FDAA-labeled and FISH-stained Gram-positive species in human-derived microbiota. Representative images of FDAA-labeled bacterial species belonging to the families Coriobacteriaceae (a,b), Clostridiaceae (c-f), Lachnospiraceae (g-j), Ruminococcaceae (k-m) and Erysipelotrichaceae (n-p) are shown. Scale bar, 2 μm. Representative photographs of bacteria, which showed consistent labeling pattern in each species from at least three independent FISH experiments, are shown

Out of the 42 FISH labeling tested, we were able to identify 32 species with high selectivity, covering bacteria from nine families, with half-stained Gram-negative (Figure 2) and the other half stained Gram-positive bacteria (Figure 3). Gram-negative bacteria that belonged to the family Bacteroidaceae and Porphyromonadaceae were 1–2 μm rods, and showed relatively weak FDAA labeling due to their thinner PGN (Figure 2(a–j)). Some Gram-negative bacteria belonging to the *Clostridium* (Figure 2k-m) and *Megamonas* (Figure 2(n–p)) genus of the phylum Firmicutes, however, showed comparable FDAA labeling intensities with many Gram-positive bacteria. This leads to the speculation that these bacteria may possess Gram-positive-like PGN structures, even though they are normally stained as Gram-negative.^13,14^ The labeling patterns of the species belonging to the *Clostridium* genus (Figure 2(k–m)) were relatively similar, which divided in binary fission with a red septum in the middle of the bacteria. *Megamonas* (Figure 2(n–p)) bacteria were large rods with rounded ends, and the presence of volutin granules^15^ could be found in some species (Figure 2n). Furthermore, the labeling signals from the two FDAA probes were highly overlapped in these three species, indicative of their highly active growth and metabolism during the labeling process.

Most of the labeled Gram-positive bacteria had higher FDAA labeling intensities than Gram-negative bacteria. The distributions of the two FDAAs among different bacterial species, which belonged to the family Coriobacteriaceae (Figure 3(a, b)), Clostridiaceae (Figure 3(c–f)), Lachnospiraceae (Figure 3(g–j)), Oscillospiraceae (Figure 3k-m) and Erysipelotrichaceae (Figure 3(n–p)), revealed their *in vivo* growth and dividing patterns correspondingly. Among these bacteria, some species worth special notes for their special morphologies. *Collinsella aerofaciens* showed an oval-shaped endospore-like structure at one end (Figure 3(b)). Because *C. aerofaciens* is not a spore-forming bacillus,^16^ these data indicate that they may reproduce by budding. Interestingly, most of the *Anaerostipes hadrus* cells exhibited asymmetric distribution of FISH signals, where only half of the cell (Figure 3(g) and Figure S5a) was FISH-stained. This suggests that most of their ribosomes were incorporated into daughter cells during division in *A. hadrus*, while the mother cells lost them. Moreover, some filamentous *A. hadrus*, which did not separate into two daughter cells during division (Figure S5b), only presented FISH signals on the two ending cells, further supporting this inference. Strong labeling at one pole of *Anaerotruncus colihominis* was observed (Figure 3(i)), indicative of its polar growth.^5^

Of special note, the three species shown in Figure 3(n–p), all of which belonged to the family Erysipelotrichaceae, showed dramatically weaker FDAA-signals than most other Gram-positive bacteria. This might be resulted from the thin PGN structures of this family, and it’s also possible that they might have a thick extracellular layer outside the cell wall, causing reduced FDAA penetration (Figure 3(n–p)). The labeling modes of several bacteria that had not been separately cultured *in vitro* were also unveiled, including *Clostridium* sp. KLE 1755 (Figure 3(e)), *Clostridiales* bacterium 1-7-47FAA (Figure 3(f)), *Lachnospiraceae* bacterium 1-4-56FAA (Figure 3(i)), *Lachnospiraceae* bacterium 3-1-57FAA-CT1 (Figure 3(j)) and *Erysipelotrichaceae* bacterium 6-1-45 (Figure 3(o)).

### STAMP recorded the PGN remodeling of Clostridium symbiosum in two cell cycles

**Figure 4.**
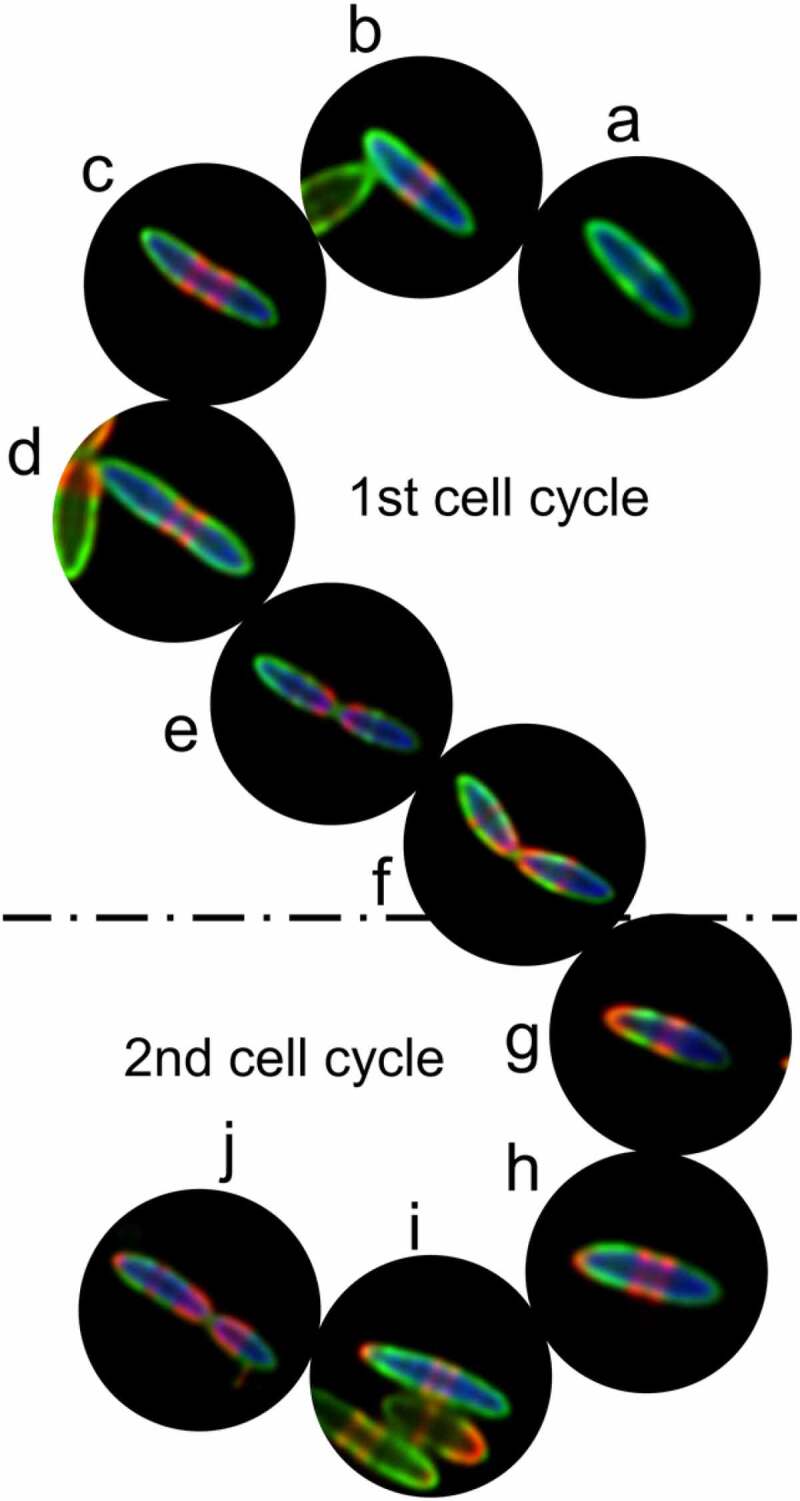
STAMP recorded the PGN construction and remodeling of *C. symbiosum* during different stages of two cell cycles. Using FISH (blue) signals, *C. symbiosum* sequentially labeled with TADA (green) and Cy5ADA (red) in the cecal microbiota were identified. Confocal images of 10 *C. symbiosum* cells at different growth stages and generations were shown to reconstruct the two division cycles of the bacteria

The capability of STAMP to chronologically record the PGN synthesis of human gut bacteria during the 6 h of labeling provides a unique opportunity to examine gut bacteria propagating at different stages of a cell division cycle. The images of *C. symbiosum*, a strict anaerobic intestinal bacterium that can cause infection in immunocompromised patients,^17,18^ are shown here as an example (Figure 4). Ten *C. symbiosum* cells with representative STAMP fluorescent labeling patterns (indicative of different generations and growth stages) in a HMA mouse gut microbiota sample were presented. Based on these images, the PGN construction and remodeling of *C. symbiosum* during two division cycles could be finely depicted.

Because different bacteria were exposed to the two FDAAs at different time, and they had asynchronized divisions and growth rates, distributions of the two colors in different *C. symbiosum* cells were very different. *C. symbiosum* presents two PGN synthesis modes: “medial” (also called “pre-septal”) elongation,^19^ which is the synthesis of PGN near the division plane before septation (Figure 4(a–d) and (g–h)), and septum formation at the division plane to complete division (Figure 4(e, f, and j)). During a division cycle, new PGN incorporation dominated at the pre-septal site to elongate the *C. symbiosum* cells, and exhibited two annular red-labeled incorporation regions (Figure 4(c, d)). When the elongasome reached approximately twice the length of the unit cell, the mother cell initiated a continuous reduction in diameter (septal invagination) at the division plane (Figure 4(e)) to start the division, and the PGN synthesis occurred at the site of septation shaped the new polar caps of the daughter cells (Figure 4(f)). Meanwhile, new PGN constructions at the midcell of daughter cells were also launched (Figure 4(f)). The resulting daughter cells which had one red-labeled pole continued to start the second division cycle (Figure 4(h–j)). Interestingly, the growth and division patterns of *C.*

*symbiosum* cultured and STAMP-labeled *in vitro* (Figure S6) were very similar with those labeled *in vivo*. Thus, by integrative analysis of the bacterial division modes and the STAMP labeling patterns of individual cells, their distinct propagating generations *in situ* could be determined, offering a unique tool for understanding the microbiology processes in the gut, and an opportunity to compare the *in vivo* and *in vitro* growth modes of the same bacteria.

### Varied morphologies of the same species in different mammalian hosts

When compared the FDAA-labeled microbiotas in HMA with the mouse natives, we noticed that the overall morphologies of gut bacteria in the human-derived microbiota were very different from the indigenous mouse microbiota. We then measured the lengths of the spindle-shaped bacteria and long bacilli, and three different bacterial species in the microbiotas from donor’s fecal, HMA mouse, and mouse native microbiotas, respectively. We found that the cellular lengths in donor’s fecal microbiota and human-derived microbiota were significantly shorter than those from the mouse native microbiota (*P* < .0001, Figure S7, S8).

**Figure 5.**
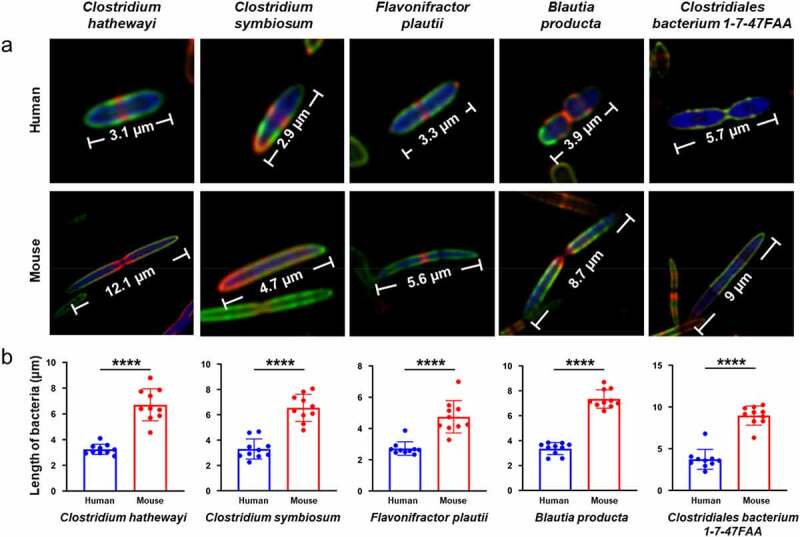
Confocal fluorescence imaging of the same bacterial species from either HMA or native mouse gut microbiota. (a) The lengths of the species (*C. hathewayi, C. symbiosum, F. plautii, B. producta*, and *C*. bacterium 1-7-47FAA) in human-derived microbiota were significantly shorter than those in the mouse indigenous microbiota. Representative photographs of bacteria, which showed consistent labeling pattern in each species from at least three independent FISH experiments, are shown. (b) Statistical analysis of the lengths of the five species in human-derived microbiota and mouse indigenous microbiota. ****P < .0001, unpaired two-tailed t-test. Mean ± s.d. are presented for n = 10

We further investigated whether the morphology and FDAA labeling pattern of the same species were the same in different hosts. *Clostridium hathewayi, C. symbiosum, Flavonifractor plautii, Blautia producta*, and *Clostridiales* bacterium 1-7-47FAA in both HMA and mouse native microbiotas were identified by FISH staining. Very similar labeling patterns and morphogenesis of the bacteria stained by the same FISH probe were observed in the mouse gut microbiota (Figure S9). Despite the very different cell lengths, the FDAA labeling patterns of each species were essentially the same (Figure 5(a)). For example, *C. hathewayi and F. plautii* divided in binary fission in both hosts, with clear red-labeled septums in the middle. When the mother cells of *B. producta* in both hosts prepared their septa for division, the daughter cells also began to synthesize new septa at the midcells. However, the cellular lengths of all five species were all significantly longer in the mouse native microbiota (Figure 5(b)).

Considering that these morphological variations might be due to the bacterial adaptations to the gastrointestinal environment in GF mice, we performed FISH staining (targeting Lachnospiraceae family, *Clostridium* genus, and *C. hathewayi* species) against the donor’s fecal microbiota to visualize the original morphologies of these microbes. The bacterial sizes and shapes in the human fecal microbiota were consistent with those seen in the HMA mouse microbiota at different taxonomic levels (Figure S10), indicating that there were no obvious morphological changes during the colonization of human fecal microbiotas in the mouse gut.

It has been reported that strains of *Lactobacillus reuteri* in the mouse gut are genetically very different from those found in humans and have evolved various genes to facilitate their adaptation to the hosts.^20,21^ It’s, therefore, reasonable to speculate that the divergent trends of genome evolution in mouse and human gut microbiota may be responsible for the varied morphologies of the same species observed in different hosts. The differences in the physiology and immunity of hosts, such as the pH values of intestine, the level of oxygen tension, and the glycan profiles of mucus, were possible to promote this host-specific adaptation. Currently, studies on gut microbiota from different hosts relies on DNA sequencing to analyze the changes in overall microbial compositions.^9,10,22^ To the best of our knowledge, this is the first report on the morphological differences of gut microbes in different mammalian hosts. This may partially explain why the physiological and pathological functions of certain gut bacteria in mouse could not be reproducible in human studies.

### Microbiome Atlas, a fluorescence imaging collection of bacterial growth and divisions

**Figure 6.**
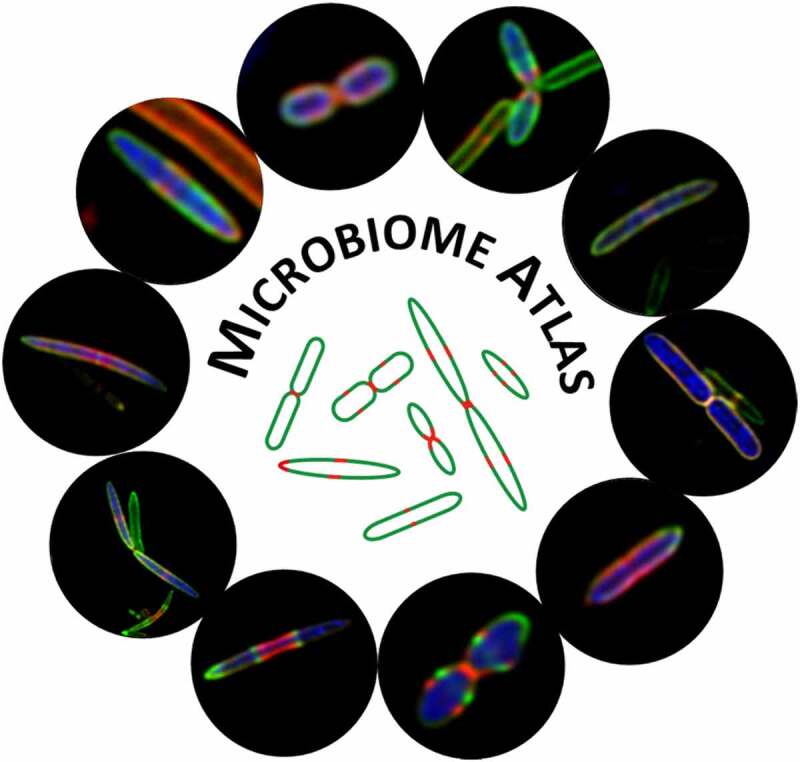
Fluorescence image library of gut microbiotas. Microbiome Atlas summarizes the fluorescence images of bacterial growth and divisions collected from microbiotas of various hosts

The successful revealing of *in vivo* growth and division patterns of human gut bacteria using STAMP+FISH protocol in HMA mouse model demonstrated the adaptability of our strategy for investigating the gut “dark matter” from a broad range of hosts. To better comprehend the vast bio-diverse phenotypes of gut bacteria and create an open-access database for facilitating communications among cellular microbiologists, we set up a Microbiome Atlas website (https://www.microbiome-atlas.com/) to integrate the

fluorescence images of microbiota obtained by STAMP + FISH labeling strategy (Figure 6). Currently, Microbiome Atlas contains detailed fluorescence images of 63 bacterial species/genus, including 33 species and 3 genera from human, 12 species and 15 genera from mouse,^5^ and 15 species from rat gut microbiota.^6^ For each species/genus, detailed descriptions and images are shown in individual page with the corresponding FISH sequences and cell sizes provided (an example shown in Figure S11). At least four signal channels including bright field, two FDAA channels, and merged fluorescence are displayed. Microbiome Atlas offers a forum to summarize our understanding of gut microbiotas. The new main query interface will soon accept imaging data submission from other researchers.

## Discussion

Our STAMP+FISH labeling strategy has been used in deciphering the basic microbiology of mouse gut microbiota.^5,23^ In this study, to better understand the microbial cytology knowledge of the human microbiota that’s highly different from the mouse native microbiota, we integrated the use of this labeling strategy with HMA mouse model for unveiling the *in vivo* growth and dividing patterns of bacteria in human microbiotas, including many unculturable species. This provides us with a unique opportunity to directly observe the microbial behaviors of human gut bacteria *in vivo*. This germ-free mice-based gut microbiota transplantation strategy paves a new way for investigating gut microbiotas in a highly diverse range of hosts, including most mammals. We further constructed an open-access database to collect the fluorescence imaging data of gut microbiotas in various hosts benefited from this imaging strategy, providing a new platform for facilitating communications among scientists in cellular microbiology and microbiota research.

Despite the distinctions in bacterial compositions between the two groups of human-derived microbiotas, there were no apparent differences in the growth and division patterns of the same bacterial species in the two groups, which further verified the results of the growth patterns of human gut bacteria obtained in our study. It could be insightful for deciphering the microbial processes when different factors, like diets, drugs, or immune factors, are applied to explore whether the growth patterns of human gut bacteria will change responsively. Furthermore, the inconsistent relative abundance of the same bacterial species identified from different metagenomic profilers (DNA-to-marker methods or DNA-to-DNA methods),^24^ endorsed the value of our FISH-based abundance assessment of bacteria in the gut microbiota. Of note, in this HMA model, a new method that we recently developed (MeDabLISH)^25^ to measure bacterial growth rates *in vivo* using their FDAA labeling and FISH staining, can also be applied. This will allow to compare the growth rates of the same bacterial species, which originally reside in the gut of different hosts, in the same mouse model. For FISH specificity verification, because of the technical challenge in finding ideal controls for each new sequence (difficulties in culturing a large variety of gut bacterial species separately *in vitro*), we introduced a soil microbiota sample, which might not be the most appropriate control.

The surprisingly different bacterial lengths between the HMA and mouse native gut microbiota bring new scopes of understanding gut bacteria. Varied adaptations of gut bacteria may cause distinct physiological/pathological roles by the same species in different hosts. The factors influencing these adaptations merit further investigations to expand our understanding of the coevolution between the gut microbiotas and their hosts. Ongoing work is being performed to assess the potential causes that lead to these morphological differences between mouse and human bacteria from multiple levels, including genetic level, epigenetic level, etc.

## Materials and methods

### Reagents

FDAA probes were bought from Chinese Peptide Company (Hangzhou, China). FISH probes used in this study were synthesized and labeled at the 5′ ends with FAM (carboxyfluorescein) by Sangon Biotech (Shanghai, China). Tryptone, peptone, calf brain infusion, beef heart infusion, yeast extract, glucose, L-cysteine, vitamin K1, vitamin K3, paraformaldehyde (PFA) and phosphate buffer saline (PBS) were purchased from Sangon Biotech (Shanghai, China). Other chemicals, not noted above, were from Sigma–Aldrich (St. Louis, MO, USA).

### Bacterial strains and culture

*Bacteroides fragilis* (ATCC 25285), *Bacteroides vulgatus* (ATCC 29327), *Bacteroides thetaiotaomicron* (ATCC 29148), *Bacteroides uniformis* (ATCC 8492) and *Bacteroides ovatus* (ATCC 8483) were purchased from American-type culture collection (Manassas, VA, US). *Clostridium symbiosum* (ATCC 14940) was kindly provided by Dr. Jingyuan Fang’s lab at Shanghai Jiao Tong University School of Medicine.

The *Bacteroides* strains were resuscitated on blood agar (BA) under anaerobic conditions (Concept 400, Baker Ruskinn, UK). After a 24 ~ 48 hours anaerobic (80% N_2_, 10% CO_2_, 10% H_2_) incubation at 37°C, at least 3 colonies were picked into tryptone-yeast extract-glucose (TYG) growth medium^26^ and grown anaerobically at 37°C for 12 ~ 24 hours. TYG medium consisted of (per 1000 ml): 10 g tryptone, 5 g yeast extract, 2 g glucose, 0.5 g L-cysteine, 100 ml of 1 M pH 7.2 KPO_4_, 40 ml of TYG salts solution (per 1000 ml: 0.5 g MgSO_4_·7H_2_O, 10 g NaHCO_3_, 2 g NaCl), 1 ml of 0.8% CaCl_2_ solution, and 1 ml of 0.4 mg/ml FeSO_4_. 1 ml of 1 mg/ml vitamin K3 solution and 1 ml of hematin-histidine solution (12 mg hematin dissolved in 10 mL 0.2 M pH8 histidine solution) were added after the medium was autoclaved. The bacterial cultures of *Bacteroides* strains were separately collected, washed with 3 × 1.5 ml PBS by centrifugation (15,000 × g, 2 min), and resuspended in sterile PBS for subsequent experiments.

*C. symbiosum* was grown overnight at 37°C under anaerobic conditions (80% N_2_, 10% CO_2_, 10% H_2_) in brain heart infusion (BHI) modified broth medium (per 1000 ml: combine 10 g peptone, 12.5 g calf brain infusion, 5 g beef heart infusion, 2 g dextrose, 5 g NaCl, 2.5 g Na_2_HPO_4_·12H_2_O, 5 g yeast extract, 5 g K_2_HPO_4_, 0.05 g L-cysteine, 1 mg resazurin sodium salt, 5 mg hemin, and 1 μl vitamin K1).

### Mice

The germ-free BALB/c mice were maintained in flexible film gnotobiotic isolators at the Department of Laboratory Animal Science at the Third Military Medical University. The mice were fed a standard autoclaved chow diet and water *ad libitum* under a strict 12 h light/12 h dark cycle and constant temperature (21–22°C) and humidity (55 ± 5%). All animal experiments were conducted in accordance with the National Institutes of Health Guide for the Care and Use of Laboratory Animals and were approved by the Ethics Committee of Third Military Medical University.

The specific pathogen-free (SPF) male C57BL/6 mice (6-week-old), obtained from Jie Si Jie Laboratory Animals (Shanghai, China), were bred at the animal facility of Renji Hospital, School of Medicine, Shanghai Jiao Tong University. The mice were housed in a specific pathogen-free environment under the same feeding conditions as those of GF mice. All animal experiments were carried out according to the guidelines approved by the Institutional Animal Care and Use Committee of the Shanghai Jiao Tong University School of Medicine.

### Fecal microbiota transplantation (FMT)

Male 6-week-old GF mice were randomly divided into two groups mice (five in each) and received stool from two healthy volunteers (donor 1 or donor 2). As described previously,^27^ each fecal sample (0.1 g) from either donor 1 or donor 2 volunteer was suspended with 1.5 ml of reduced sterile PBS. After thorough mixing, an aliquot of 200 μl of fecal suspensions was administered to each GF mouse by gavage twice (at week 0) to generate the HMA mouse model. After colonization, the two group of HMA mice were housed in different gnotobiotic cages for 3 weeks to prevent cross-contamination of microbiotas.

### STAMP labeling of human-derived or mouse native microbiotas with FDAA probes

After successful transplantation of human microbiotas, the HMA mice sequentially received two different FDAA probes (200 μl, 1 mM TADA or Cy5ADA in sterile H_2_O) by gavage at an interval of 3 h to label the cell wall of the microbiotas. The cecal microbiotas of HMA mice were collected using a previously published method.^5,23^ Briefly, the cecum of HMA mice was dissected separately, finely cut with a pair of iris scissors in 2 ml of PBS, and then filtered with a sterile cell strainer (40 μm) to remove the tissue debris. The microbiotas from the five HMA mice in each group were combined and thoroughly mixed for subsequent FISH experiments. The human-derived microbiotas were washed with 3 × 1.5 ml PBS by centrifugation (15,000 × *g*, 3 min) and then resuspended in 1 ml PBS for subsequent experiments. The STAMP-labeled cecal microbiota of traditional SPF mice were collected using the same protocol.

### In vitro culture of soil microbiota

Five grams of soil collected from the Tangqiao Park in Shanghai were homogenously resuspended in 50 ml of sterile physiological water. Ten-fold serial dilutions of the soil suspension were performed to 10^−3^. One hundred microliters of the 10^−3^ dilutions were dispersed on beef extract-peptone agar medium (per 1000 ml: containing 3 g beef extract, 10 g peptone, 5 g sodium chloride, 20 g agar, pH 7.2 ± 0.2) and then incubated at 37°C for 24 ~ 48 hours under aerobic conditions. Soil bacterial cultures were collected, washed with 3 × 1.5 ml PBS by centrifugation (15,000 × g, 2 min), and resuspended in sterile PBS for subsequent experiments.

### FISH probe design

The oligonucleotide probes used to target species were either identified in previous reports or designed de novo using an algorithm previously reported^5^ and listed in Table S4 and Table S5. Since probes with high stability of secondary structures are difficult to hybridize with the 16S sequences, candidate probes with low-free energy should be avoided. In this study, we further optimized the pdesign, an algorithm developed in a previous work,^5^ to prevent the formation of secondary DNA structures. In detail, RNAfold,^28^ a high throughput DNA/RNA secondary structure prediction method, was integrated and the free energy of each candidate was printed in the result file to instruct the user to select the most appropriate probe. The code of this new version of pdesign can be found in https://github.com/songjiajia2018/pdesign-v2.0/. The newly designed probes were named in accordance with previously reported rules.^29^

### Specificity confirmation of FISH probes

The labeling specificities of the newly designed FISH sequences were evaluated using methods described previously.^5^ EUB338 and NONEUB probes were used as the positive and negative controls, respectively. The FISH probes were separately tested against a soil microbiota sample that didn’t share any genera with the human-derived microbiotas, using FISH protocols described below. No labeling against the soil microbiota sample was observed in any of the 32 FISH probes presented in Table S4 in the test (Figure S12).

Further specificity confirmation tests of the newly designed FISH probes were also performed from multiple aspects using the previously published methods.^5^ Bacterial species tagged with new FISH probes were analyzed by confocal fluorescence microscopy to assess whether the cell morphologies and labeling patterns of the species stained by the same probe were consistent (Fig. S3). Flow cytometry was carried out to analyze whether the labeling ratios of bacterial species tagged with new FISH probes in the microbiota was consistent with their relative abundances in sequencing (Fig. S4).

In order to verify that the FISH probe targeting the Bacteroides species of interest does not bind *in vitro* against other species in the same genera, eight FISH probes were separately tested against five samples of *Bacteroides* strains, using FISH protocols described below. The eight FISH probes of *Bacteroides* species bound to the species of interest, but not to other species in the same genera (Figure S13), further confirming the specificities of the eight FISH probes.

### Fluorescence in situ hybridization

FISH experiments were performed according to an approach adopted from a previous report.^5^ Briefly, microbiotas were first fixed in 2% PFA in PBS (v/v) at room temperature for 1.5 h, and then washed twice with PBS. An equal volume of absolute ethanol was added into the bacterial suspensions in PBS, and stored at −30°C until use.

Because the gut microbiota samples were labeled with FDAA probes containing TAMRA and Cy5, the FISH probes in Tables S4 and Tables S5 were synthesized and labeled at the 5ʹ ends with FAM. Before FISH staining, the microbiota samples of each group were transferred into small aliquots. After washed with PBS, the bacterial pellets were resuspended in a hybridization buffer [0.9 M NaCl, 20 mM Tris (pH 7.5), 0.01% SDS, and formamide, if required] (Table S4 and Table S5). Then, each FISH probe was added with a final concentration of 5 ng/l into corresponding tube separately and incubated overnight at indicated temperature (Table S4 and Table S5) using a ThermoMixer (Eppendorf, Hamburg, Germany). After hybridization, each microbial sample was then washed twice with washing buffer (0.9 M NaCl, 20 mM Tris, pH 7.5, 0.01% SDS) for 15 min at the respective washing temperatures. Bacteria were then resuspended in PBS for analysis with fluorescence microscopy and flow cytometry.

### Growth and division of C. symbiosum in vitro

*C. symbiosum* was grown overnight in BHI modified broth medium at 37°C. TADA was added to the medium to a final concentration of 500 µM for 3 h labeling, and then removed by centrifugation (15,000 × *g*, 2 min). Afterward, the *C. symbiosum* was labeled with Cy5ADA at a final concentration of 500 µM for 1 h, 2 h, and 3 h, respectively. The above operations were performed under anaerobic conditions. Finally, the three groups of bacteria were collected, washed with 3 × 1.5 ml PBS by centrifugation (15,000 × g, 2 min), and resuspended in sterile PBS to reach an appropriate concentration for confocal fluorescence imaging.

### Confocal fluorescence microscopy

Labeled bacteria were inoculated onto agarose pads (1.5% w/v in PBS, ~1 mm thick) on slides and covered with glass coverslips. Confocal fluorescence imaging of bacterial samples was performed on a laser scanning confocal microscope (Leica TCS SP8, Solms, German) using a 63× oil immersion objective (HC PL APO CS2, NA 1.40). A 488 nm laser was used to excite FAM, a 552 nm to excite TAMRA, and a 638 nm to excite Cy5. Strong FISH (FAM) signals were used for taxonomic identification of the corresponding bacteria, the FDAA signals of which were then recorded and used for growth pattern analysis. The internal Leica HyD detector was used to detect the emission of each fluorophore. Images were obtained using the Leica Application Suite Interface (LAS X) software. Deconvolution of the images were processed using Huygens Essential Deconvolution software (Scientific Volume Imaging B.V., Hilversum, Netherlands) integrated in Leica LAS-X software, with a maximum of 40 iterations.

### Flow cytometry

Based on a previously published method,^25^ flow cytometry was performed to analyze the labeling rate of FISH-stained bacterial species in the human-derived microbiotas. A 2.5 μl aliquot of stained bacterial suspension was added to 200 μl sterile PBS in fresh round-bottom polystyrene tube. Tubes were vortexed to resuspend the bacteria and then run at the lowest flow rate on a CytoFLex flow cytometer (Beckman Coulter Life Sciences, Indianapolis, IN, US) until approximately 15,000 events were recorded. Analysis and gating for FISH-labeled bacteria were performed using CytExpert software and FlowJo (V 10.0.8r1).

### 16S rDNA sequencing

DNA of the microbiotas from human feces or HMA mice were extracted either using the stool DNA Kit or bacterial DNA Kit (Omega Bio-Tek, Norcross, GA, USA) following manufacturer’s instructions, respectively. The 16S rRNA gene sequencing analysis of the microbiota composition before and after FMT was performed by Realbio Genomics Institute (Shanghai, China). The V3–V4 hypervariable regions of the 16S ribosomal RNA genes were amplified by PCR (95°C for 3 min, followed by 30 cycles at 98°C for 20 s, 58°C for 15 s, and 72°C for 20 s and a final extension at 72°C for 5 min) using barcoded primers 341F 5ʹ-CCTACGGGRSGCAGCAG-3ʹ and 806R 5ʹ-GGACTACVVGGGTATCTAATC-3ʹ. PCR reactions were performed in 30 μl mixture containing 15 μl of 2 × KAPA Library Amplification ReadyMix, 1 μl of each primer (10 μM), 50 ng of template DNA and ddH_2_O. Amplicons were extracted from 2% agarose gels and purified using the AxyPrep DNA Gel Extraction Kit (Axygen Biosciences, Union City, CA, US) according to the manufacturer’s instructions and quantified using Qubit®2.0 (Invitrogen, US). All quantified amplicons were pooled to equalize concentrations for sequencing using Illumina MiSeq/HiSeq (Illumina, Inc., CA, USA). The paired end reads of 250 bp were overlapped on their 3 ends for concatenation into original longer tags by using PANDAseq (https://github.com/neufeld/pandaseq, version 2.9).

Assembled tags, trimmed of barcodes and primers, were further checked on their rest lengths and average base quality. 16S tags were restricted between 220 bp and 500 bp such that the average Phred score of bases was no worse than 20 (Q20) and no more than 3 ambiguous N. The copy number of tags was enumerated and redundancy of repeated tags was removed. Only the tags with frequency more than 1, which tend to be more reliable, were clustered into OTUs, each of which had a representative tag. Operational Taxonomic Units (OTUs) were clustered with 97% similarity using UPARSE (http://drive5.com/uparse/) and chimeric sequences were identified and removed using Usearch (version 7.0.1090). Each representative tag was assigned to a taxon by RDP Classifer (http://rdp.cme.msu.edu/) against the RDP database (http://rdp.cme.msu.edu/) using confidence threshold of 0.8. OTU profiling table and alpha diversity analyses were also achieved by python scripts of QIIME (version 1.9.1).

### Metagenomic sequencing

DNA extraction was conducted as described above. The metagenomic sequencing analysis of the species composition from the human-derived microbiota was performed by Realbio Genomics Institute (Shanghai, China). Following the Illumina TruSeq DNA Sample Prep v2 Guide (Illumina, Inc., San Diego, CA, USA), the DNA libraries with approximately 500 bp insert sizes were constructed for each sample. The quality of all libraries was evaluated using an Agilent 2100 bioanalyzer (Agilent Technologies, Wokingham, UK) and the Agilent 2100 DNA 1000 kit. All samples were subject to 150 bp paired-end sequencing on an Hiseq X-ten platform (Illumina, Inc., San Diego, CA, USA).

Illumina raw reads were screened according to the following criteria: (1) remove adaptor contamination reads; (2) reads containing more than three ambiguous N bases were removed; (3) reads containing low quality (Q < 20) bases were trimmed; (3) reads containing less than 60% of high-quality bases (Phred score ≥20) were deleted. Then, clean reads were subjected to bacterial genomes from the National Center for Biotechnology Information GenBank with SOAPaligner (version 2.21) and reads mapped to the host genome were abandoned. The subsequent reads were selected for further analysis.

Clean reads were aligned to the NCBI database (National Center for Biological Information, http://www.ncbi.nlm.nih.gov) for the detection of known bacteria, fungi, viruses, and archaea by MetaPhlAn2 (version 2.5.0, DNA-to-marker methods) or SOAPaligner (version 2.21, DNA-to-DNA methods). Then, the aligned reads were classified as Kingdom, Phylum, Class, Order, Family, Genus, Species to count classification and abundance, and generated taxonomic relative abundance profile at different levels.

## Supplementary Material

Supplemental Material

## Data Availability

The 16S rDNA sequencing data of the human fecal microbiotas from the two donors and the HMA mouse microbiotas have been deposited in the Sequence Read Archive with BioSample accessions SAMN19653990, SAMN19653991, SAMN19653992, and SAMN19653993, respectively. The metagenomic sequencing data of the HMA mouse microbiotas from donor 1 and donor 2 have also been deposited with BioSample accessions SAMN19655720 and SAMN19655721, respectively.
