# Supplementary material for: Imaging the in vivo growth patterns of bacteria in human gut Microbiota
Source: Gut Microbes. 2021 Aug 24;13(1):1960134. doi: 10.1080/19490976.2021.1960134 (PMC8386752; doi:10.1080/19490976.2021.1960134)
Supplement: Supplemental Material [file KGMI_A_1960134_SM0355.zip › Supplementary information/Human Gut Microbiota atlas_SI_061021.pdf]

Supplementary Materials

Imaging the *in vivo* Growth Patterns of Bacteria in Human Gut Microbiota

Liyuan Lin[a#], Jia Song[a#], Jian Li[e#], Xiaolei Zuo[a], Hong Wei[b,d*], Chaoyong Yang[a,c*], Wei Wang[a*]

[a] Institute of Molecular Medicine, Renji Hospital, Shanghai Jiao Tong University School of Medicine, Shanghai, 200127, China
[b] Central Laboratory, Clinical Medicine Scientific and Technical Innovation Park, Shanghai Tenth People's Hospital, Tongji University, Shanghai 200435, China
[c] The MOE Key Laboratory of Spectrochemical Analysis and Instrumentation, Key Laboratory for Chemical Biology of Fujian Province State Key Laboratory of Physical Chemistry of Solid Surfaces, Department of Chemical Biology, College of Chemistry and Chemical Engineering, Xiamen University, Xiamen, 361005, China
[d] State Key Laboratory of Agricultural Microbiology, College of Animal Sciences and Technology, Huazhong Agricultural University, Wuhan 430070, P. R. China
[e] Institute of Immunology, PLA, Third Military Medical University, Chongqing, 400038, China

Correspondence: weihong63528@163.com; cyyang@xmu.edu.cn; wwang@shsmu.edu.cn

[#]These authors contributed equally to this work.

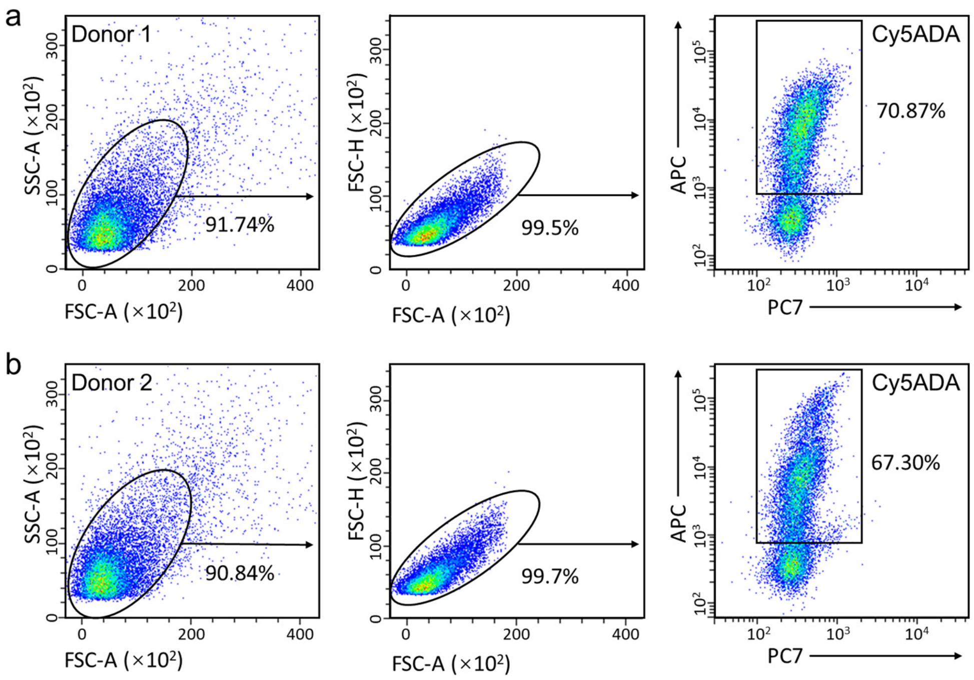

Figure S1. FDAA-labeling coverages of the two groups of human-derived microbiotas. The donor 1 group **(a)** and donor 2 group **(b)** showed comparably high FDAA labeling coverages (70.87% and 67.30%). The cecal microbiotas of the two groups of HMA mice were collected, combined separately, and analyzed by flow cytometry. Gating strategy used for labeling coverage assay is shown.

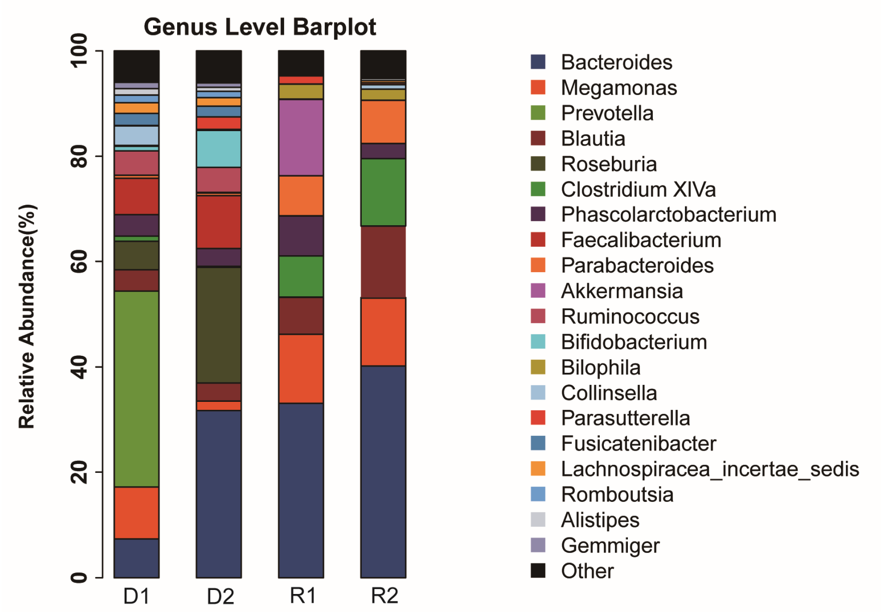

Figure S2. Taxonomic distributions of the human fecal and HMA mouse cecal microbiotas. The human fecal microbiotas from the two donors (donor 1 and donor 2, D1 and D2) and HMA cecal microbiotas from the two groups of recipient mice (donor 1 and donor 2 group, R1 and R2) were collected and analyzed by 16S rRNA gene sequencing, respectively. The top 20 bacterial genera are displayed.

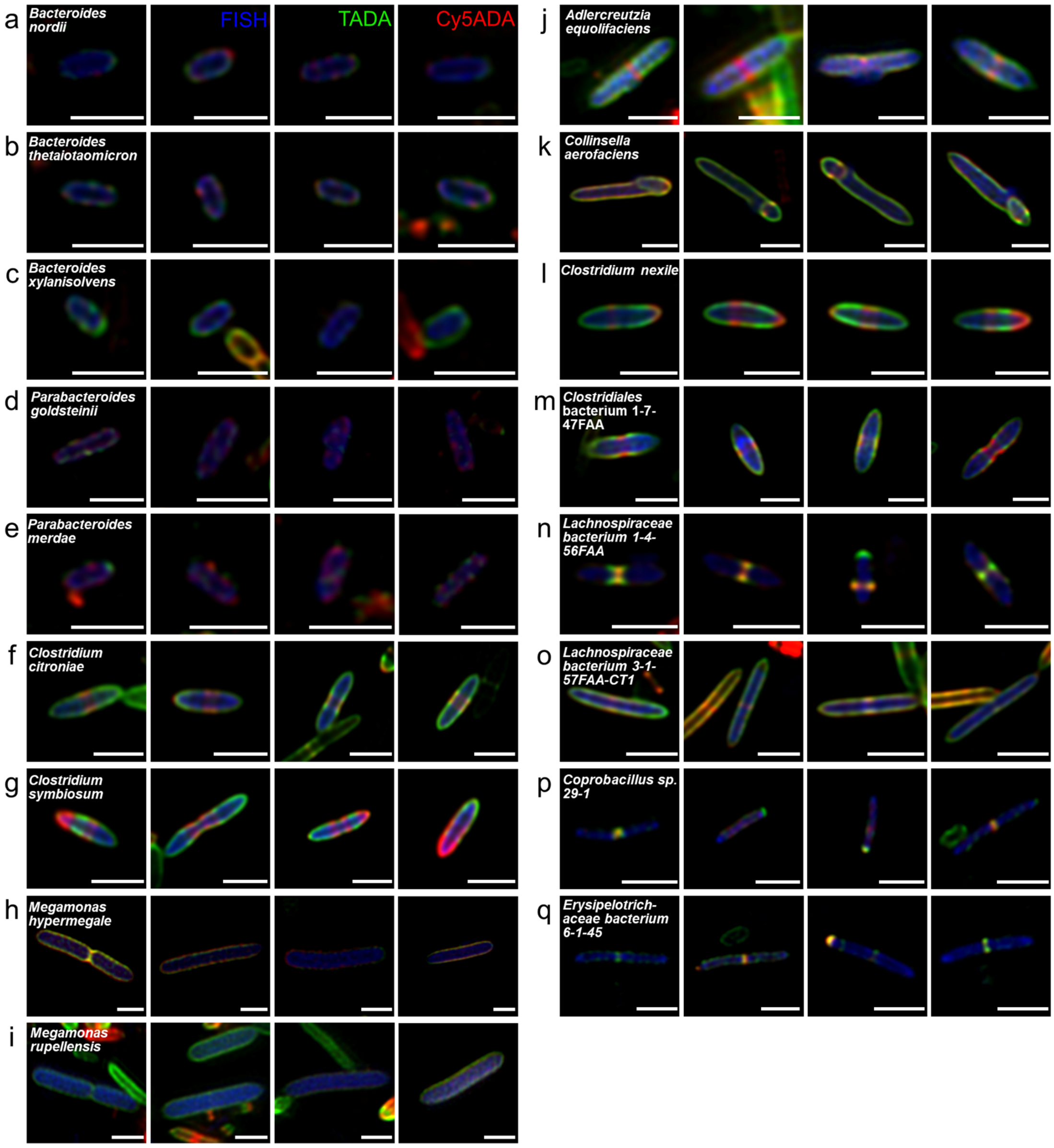

Figure S3. Confocal fluorescence imaging of each FISH probe-tagged bacterial species from the two human-derived microbiota samples showed consistent FDAA-labeling patterns and cell morphologies in each species. The human-derived microbiotas labeled with TADA (green) and Cy5ADA (red) were stained by corresponding FISH probes (blue) targeting corresponding species, and imaged by confocal fluorescence microscopy. Consistent cell morphologies and FDAA-labeling patterns of each species were observed in the two human-derived microbiota samples, including *Bacteroides nordii* (a), *Bacteroides thetaiotaomicron* (b), *Bacteroides xylanisolvens* (c), *Parabacteroides goldsteinii* (d), *Parabacteroides merdae* (e), *Clostridium citroniae* (f), *Clostridium symbiosum*(g), *Megamonas hypermegale* (h), *Megamonas rupellensis* (i), *Adlercreutzia equolifaciens* (j), *Collinsella aerofaciens* (k), *Clostridium nexile* (l), *Clostridiales* bacterium 1-7-47FAA (m), *Lachnospiraceae* bacterium 1-4-56FAA (n), *Lachnospiraceae* bacterium 3-1-57FAA-CT1 (o), *Coprobacillus* sp. 29-1 (p), and *Erysipelotrichaceae* bacterium 6-1-45 (q). Scale bars, 2 µm.

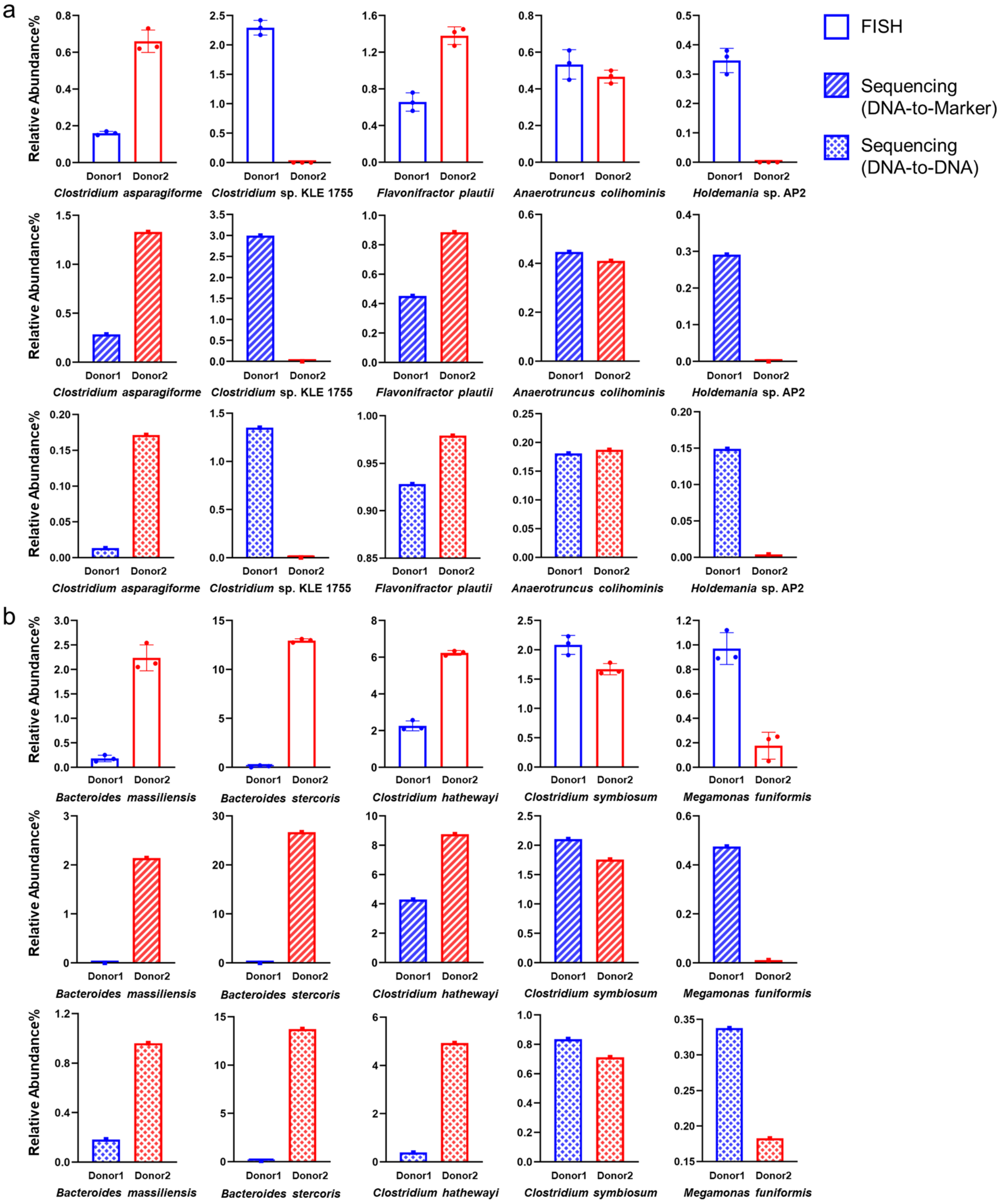

Figure S4. The labeling ratios by the newly designed FISH probes were consistent with the relative abundances of the corresponding species determined by metagenomic sequencing. In both donor 1 and donor 2 groups, the relative abundances of the five Gram-positive **(a)** and five Gram-negative bacterial species **(b)** quantified by FISH-staining were consistent with their relative abundances determined by metagenomic sequencing results. Metagenomic sequencing data were produced by two methods for classification of metagenomics species, including DNA-to-Marker methods and DNA-to-DNA methods.[1] Representative data from at least three independent FISH experiments of the human-derived microbiota samples are shown.

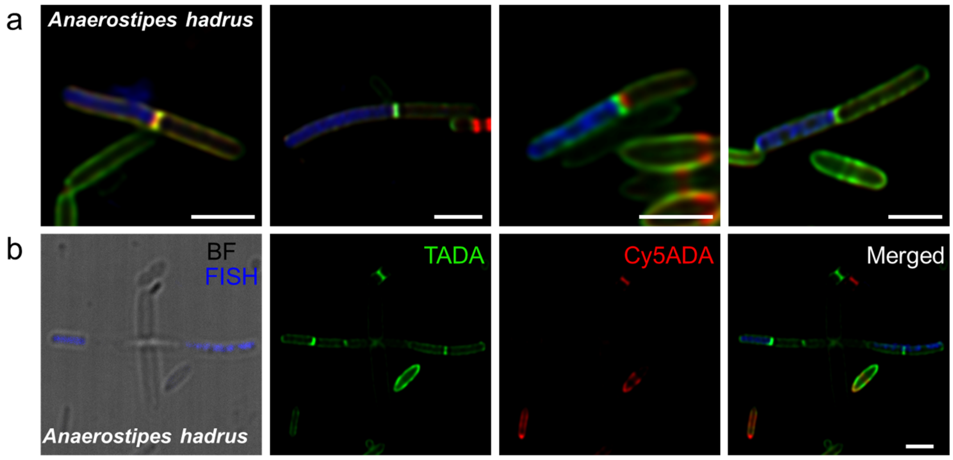

Figure S5. Confocal fluorescence imaging of *Anaerostipes hadrus*. The cecal microbiotas collected from HMA mice which received sequential labeling of TADA (green) and Cy5ADA (red) were stained by FISH probes (blue) targeting *A. hadrus*. Scale bars, 2 μm. Representative photographs of bacteria from at least three independent FISH experiments are shown.

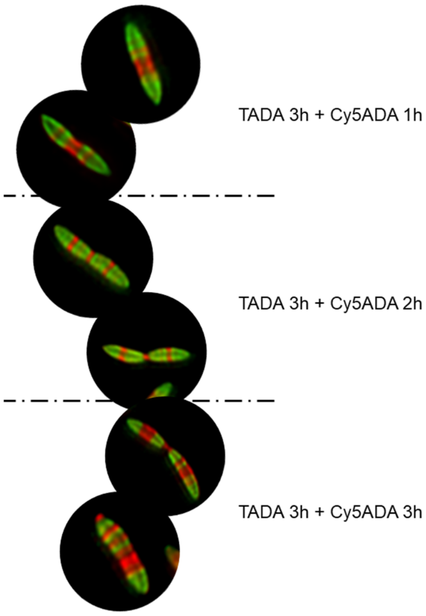

Figure S6. STAMP recorded the PGN construction and remodeling of *C. symbiosum in vitro*. *C. symbiosum* cells sequentially labeled with TADA (green) for 3 h and Cy5ADA (red) for another 1 h, 2 h and 3 h respectively, were analyzed by confocal microscopy. Representative photographs of bacteria from at least three independent FISH experiments are shown.

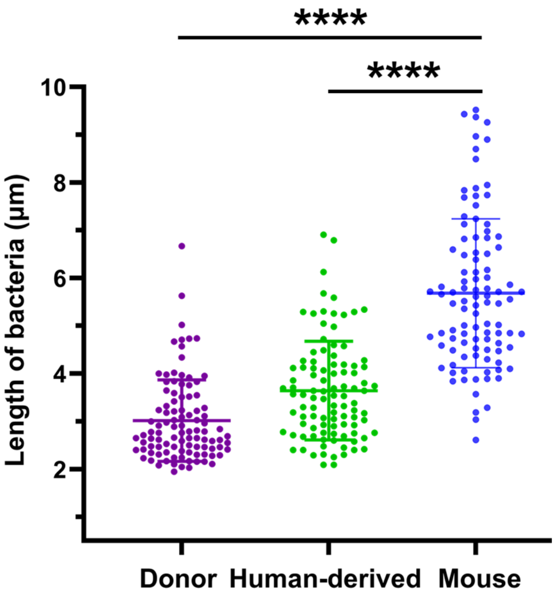

Figure S7. Statistical analysis of the lengths of spindle-shaped bacteria and long bacilli in different microbiotas. The spindle-shaped bacteria and long bacilli in donor's fecal and human-derived microbiotas were significantly shorter than those in the mouse native microbiotas. ****P < 0.0001, unpaired two-tailed t-test. Mean ± s.d. are presented for n=100.

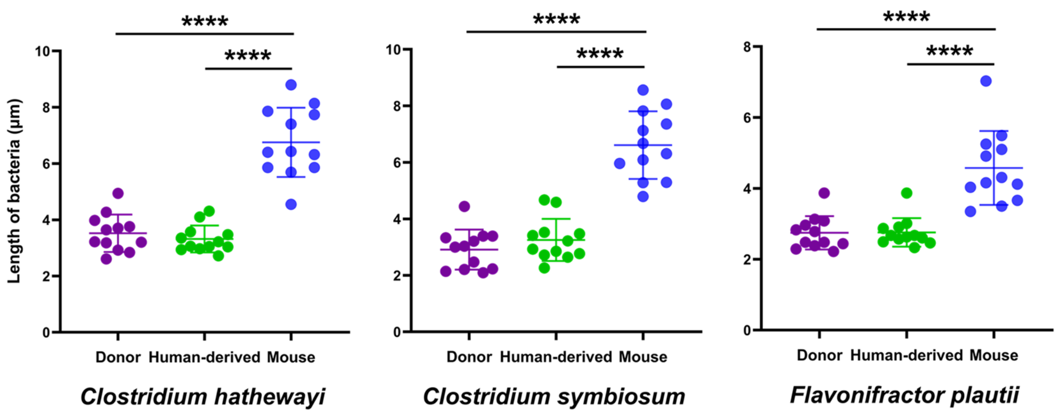

Figure S8. Statistical analysis of bacterial lengths of three species found in different microbiotas. Three species in donor's fecal and human-derived microbiotas were significantly shorter than those in the mouse native microbiotas. ****P < 0.0001, unpaired two-tailed t-test. Mean ± s.d. are presented for n=12.

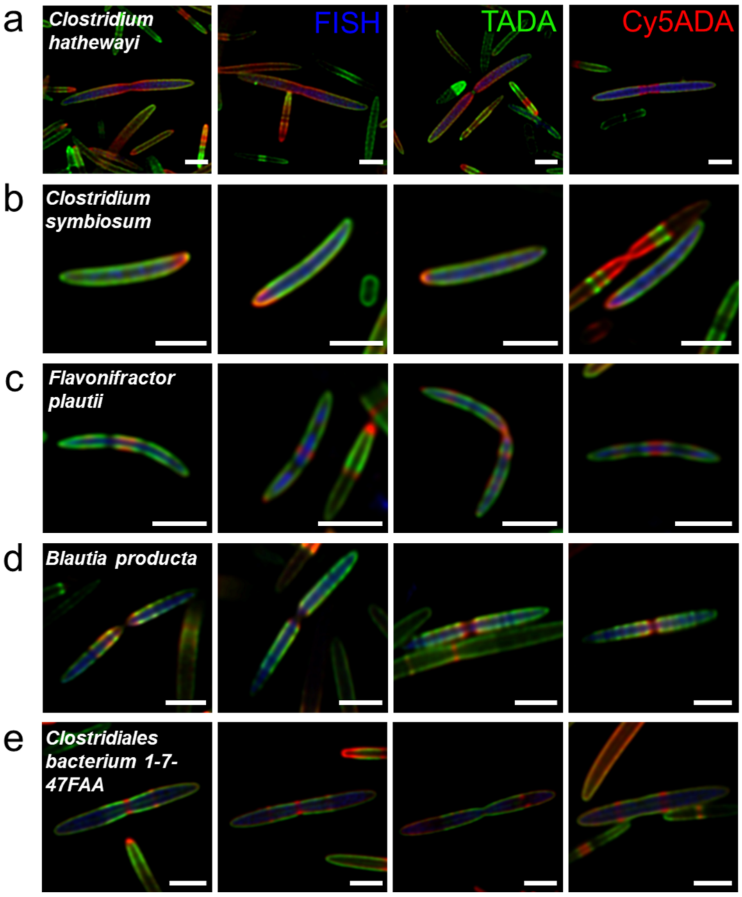

Fig. S9. Confocal fluorescence imaging of the FISH-stained mouse native cecal microbiotas showed consistent cell morphologies and FDAA-labeling patterns in each species. The mouse native microbiotas labeled with TADA (green) and Cy5ADA (red) were stained by corresponding FISH probes (blue) targeting corresponding species, and imaged by confocal fluorescence microscopy. Consistent cell morphologies and FDAA-labeling patterns were observed in each species, including *C. hathewayi* (a), *C. symbiosum* (b), *F. plautii* (c), *B. producta* (d) and *C.* bacterium 1-7-47FAA (e). Scale bar, 2 μm.

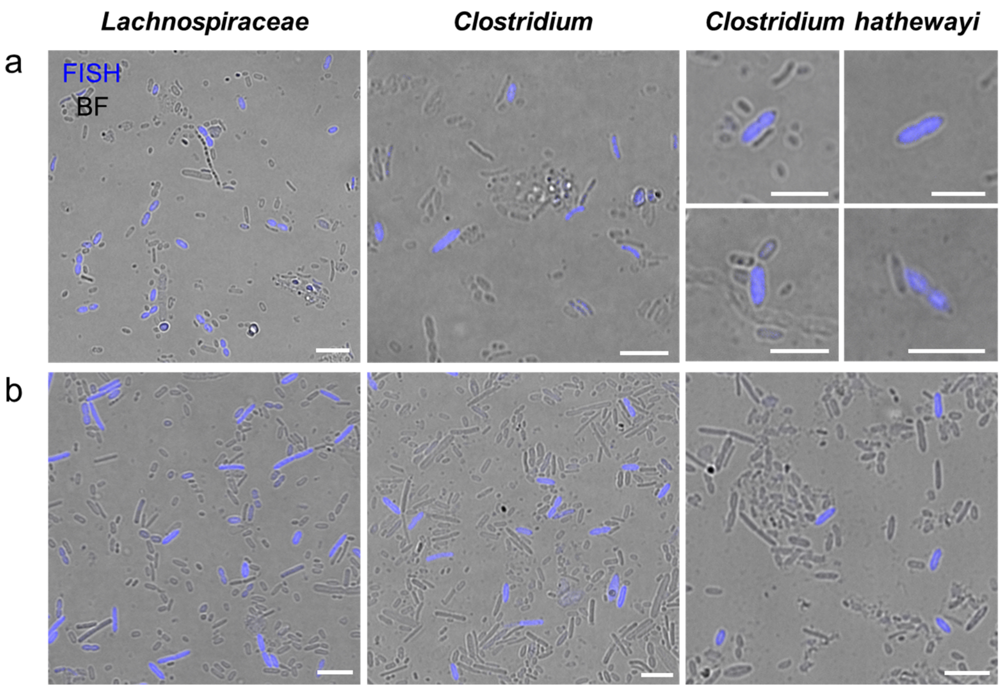

Figure S10. Confocal fluorescence imaging of the donor's fecal microbiotas and human-derived microbiotas. The sizes and shapes of bacteria from the donor's fecal microbiotas **(a)** stained by corresponding FISH probes (blue), were consistent with those from human-derived microbiotas **(b)** at different taxonomic levels, including *Lachnospiraceae* family, *Clostridium* genus, and *Clostridium hathewayi* species. Representative data from at least three independent experiment are shown. Scale bars, 5 μm.

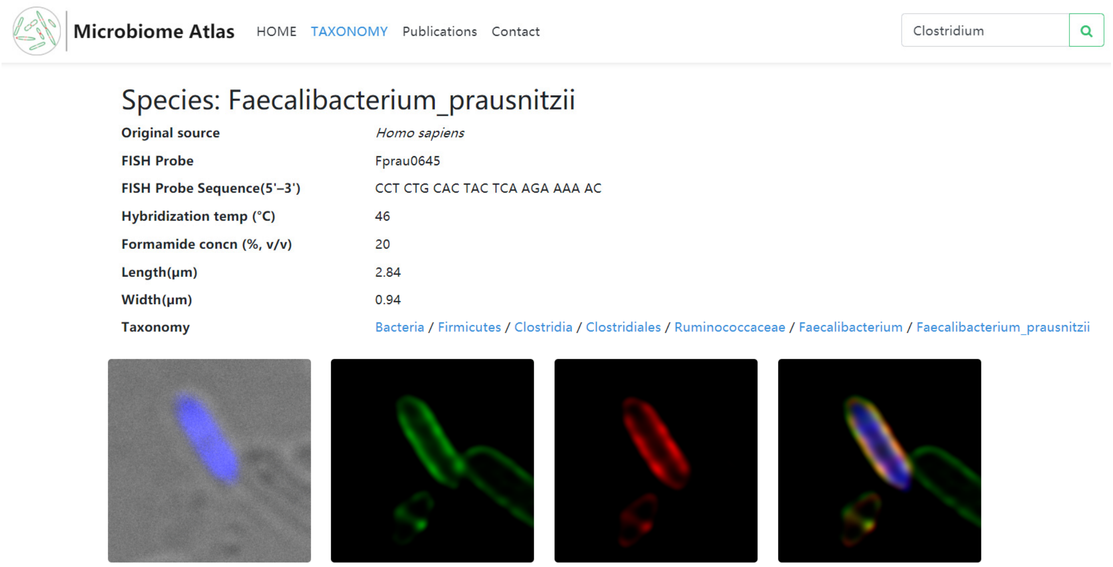

Figure S11. Detailed descriptions and fluorescence images data of *Faecalibacterium prausnitzii* in the Microbiome Atlas website. The FISH probe sequence, hybridization reaction conditions, cell sizes and fluorescence images (including, from left to right, bright field, TADA, Cy5ADA and merged) are shown in an individual page.

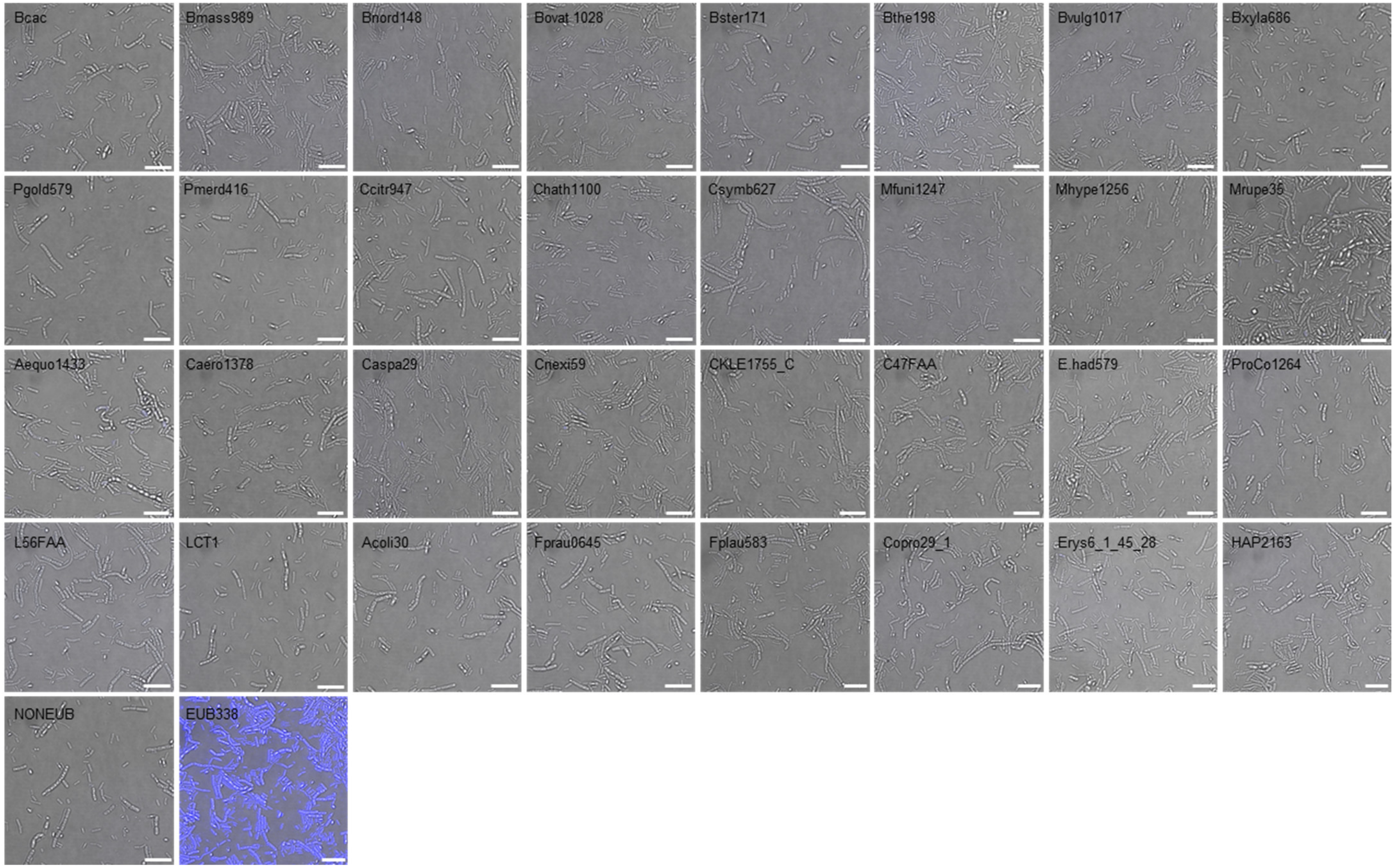

Fig. S12. None of the 32 FISH probes non-specifically bound to a soil microbiota sample. The soil microbiota from an *in vitro* culture was used to evaluate the specificity of the newly designed FISH probes. EUB338 and NONEUB probes were used as the positive and negative controls, respectively. Scale bar, 10 µm.

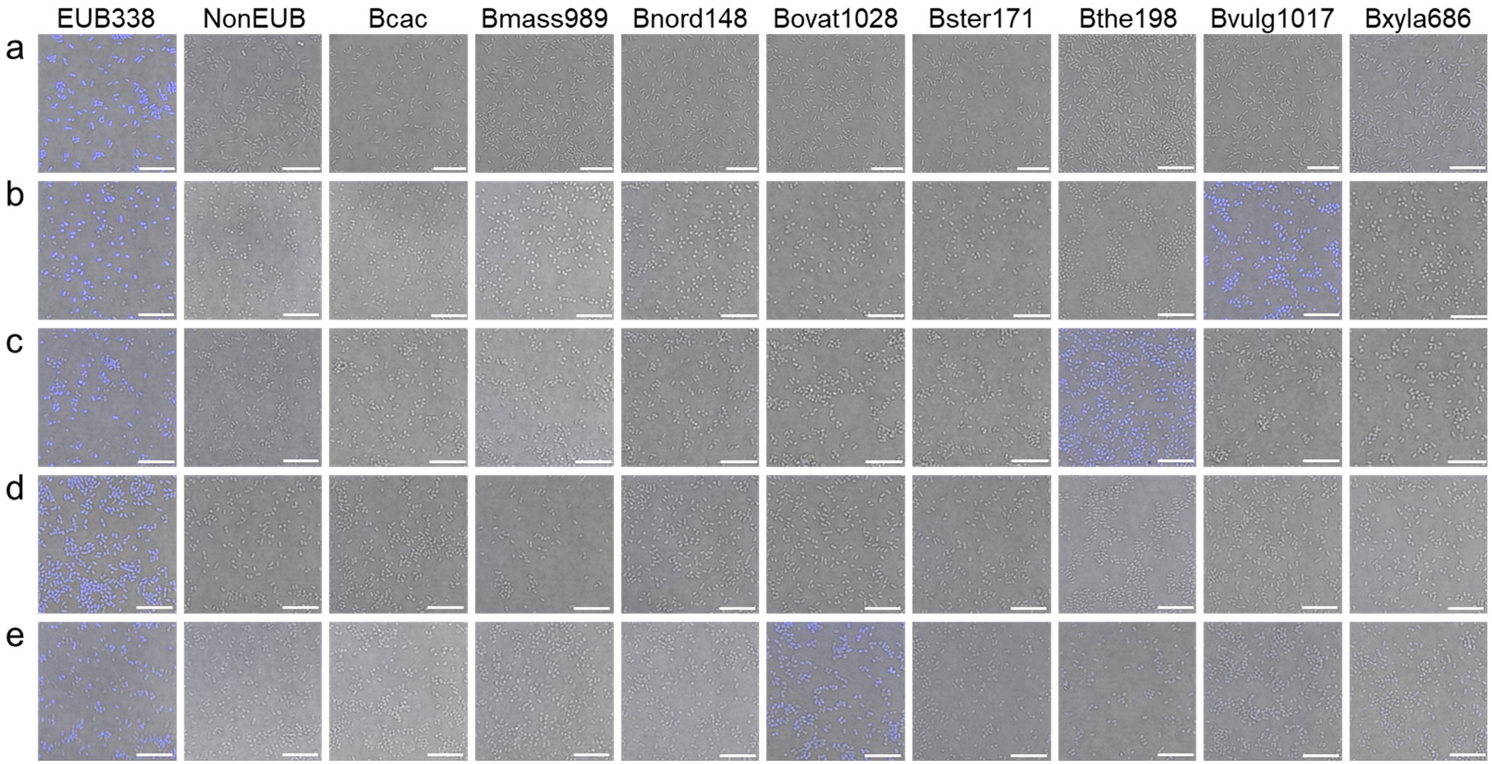

Fig. S13. Eight FISH probes targeting *Bacteroides* species specifically bound to the species of interest and not to other species in the same genera. *Bacteroides* strains cultured *in vitro*, including *Bacteroides fragilis* (a), *Bacteroides vulgatus* (b), *Bacteroides thetaiotaomicron* (c), *Bacteroides uniformis* (d) and *Bacteroides ovatus* (e), were used for assessing the specificity of FISH probes that bind to the corresponding *Bacteroides* species. EUB338 and NONEUB probes were used as the positive and negative controls, respectively. Scale bar, 10 μm.

Table S1. Taxonomic distributions of the two groups of human-derived microbiotas determined by metagenomic sequencing.

[a] Bacterial species with relative abundances <0.1% are not displayed.

Bacteria Name (Species Level)	Donor1	Donor2	Bacteria Name (Species Level)	Donor1	Donor2
Bacteroides cellulosilyticus	20.59%	0.01%	*Bacteroides nordii*	0.22%	0.00%
Bacteroides uniformis	16.47%	9.68%	*Parasutterella excrementihominis*	0.21%	0.00%
Clostridium bolteae	6.38%	8.44%	*Bacteroides ovatus*	0.16%	3.04%
Bacteroides caccae	5.79%	0.00%	*Burkholderiales* bacterium 1-1-47	0.16%	0.00%
Clostridium hathewayi	4.29%	8.75%	*Megamonas rupellensis*	0.14%	0.00%
Akkermansia muciniphila	3.63%	0.00%	*Alistipes shahii*	0.11%	0.16%
Parabacteroides distasonis	3.58%	3.87%	*Clostridium citroniae*	0.11%	0.04%
Clostridium sp. KLE 1755	3.00%	0.00%	*Blautia producta*	0.10%	0.06%
Bacteroides thetaiotaomicron	2.86%	4.42%	*Megamonas hypermegale*	0.10%	0.06%
Bacteroides vulgatus	2.39%	1.02%	*Holdemania filiformis*	0.08%	0.20%
Helicobacter bilis	2.27%	0.47%	*Adlercreutzia equolifaciens*	0.07%	0.11%
Clostridium symbiosum	2.10%	1.76%	*Erysipelotrichaceae* bacterium 6-1-45	0.06%	0.29%
Bacteroides xylanisolvens	1.89%	1.09%	*Bacteroides stercoris*	0.01%	26.70%
Bacteroides fragilis	1.79%	0.00%	*Collinsella aerofaciens*	0.00%	0.10%
Ruminococcus gnavus	1.65%	0.06%	*Bacteroides massiliensis*	0.00%	2.14%
Coprobacillus sp. 29-1	0.99%	0.00%	*Odoribacter splanchnicus*	0.00%	0.23%
Bilophila wadsworthia	0.88%	1.08%	*Parabacteroides goldsteinii*	0.00%	0.32%
Eubacterium cylindroides	0.59%	0.00%	*Parabacteroides merdae*	0.00%	1.62%
Faecalibacterium prausnitzii	0.57%	0.01%	*Alistipes putredinis*	0.00%	1.63%
Clostridiales bacterium 1-7-47FAA	0.51%	1.82%	*Clostridium nexile*	0.00%	0.31%
Megamonas funiformis	0.48%	0.01%	*Clostridium* sp. ATCC BAA 442	0.00%	1.49%
Flavonifractor plautii	0.45%	0.89%	*Lachnospiraceae* bacterium 1-4-56FAA	0.00%	0.26%
Anaerotruncus colihominis	0.45%	0.41%	*Lachnospiraceae* bacterium 3-1-57FAA-CT1	0.00%	2.85%
Holdemania sp. AP2	0.29%	0.00%	*Subdoligranulum* sp. 4-3-54A2FAA	0.00%	0.36%
Clostridium asparagiforme	0.28%	1.33%	*Phascolarctobacterium succinatutens*	0.00%	1.84%
Lachnospiraceae bacterium 7-1-58FAA	0.28%	0.25%	*Desulfovibrio piger*	0.00%	0.15%

Table S2. Taxonomic distributions of the donor's fecal microbiotas and the HMA mouse microbiotas determined by metagenomic sequencing.

Bacteria Name (Species Level)	Donor1_Donor	Donor1_Receptor	Donor2_Donor	Donor2_Receptor
Prevotella	35.6628%	0.0035%		
Megamonas	9.4586%	10.2491%	1.5917%	10.8359%
Bacteroides	7.0412%	25.9443%	26.7383%	33.8604%
Faecalibacterium	6.7039%	0.0316%	8.5485%	0.0105%
Roseburia	5.1755%	0.0457%	18.5060%	0.0035%
Ruminococcus	4.4166%	0.0000%	4.0160%	0.0000%
Phascolarctobacterium	3.9106%	5.9098%	2.8319%	2.4068%
Blautia	3.8685%	5.4636%	2.8811%	11.3981%
Collinsella	3.5522%	0.0176%		
Fusicatenibacter	2.1960%	0.0000%	1.7146%	0.0141%
Lachnospiracea-incertae-sedis	1.9325%	0.0562%	1.3879%	0.3408%
Megasphaera	1.4511%	0.0000%		
Romboutsia	1.3984%	0.0000%	1.0049%	0.0000%
Alistipes	1.1911%	0.0211%	0.6430%	0.3408%
Gemmiger	1.1314%	0.1827%	0.6992%	0.0000%
Ruminococcus2	0.9908%	0.1897%	0.0141%	0.4989%
Clostridium XIVa	0.9381%	6.1066%	0.1581%	10.8429%
Bifidobacterium	0.8433%	0.0422%	5.9239%	0.0141%
Sutterella	0.5622%	0.0000%		
Parabacteroides	0.5551%	6.0188%	0.4041%	6.9042%
Dorea	0.4954%	0.1897%	0.3549%	0.6816%
Anaerostipes	0.4708%	0.5797%	0.6992%	0.0386%
Oscillibacter	0.2565%	0.2811%	0.2214%	0.4638%
Mitsuokella	0.2108%	0.0000%		
Desulfovibrio	0.1687%	0.0000%		
Bilophila	0.1476%	2.2206%	0.1581%	1.7568%
Fusobacterium	0.1441%	0.0000%		
Escherichia/Shigella	0.1370%	0.0386%	0.0808%	0.0632%
Butyricicoccus	0.1335%	0.4181%	0.0843%	0.2811%
Asaccharobacter	0.1019%	0.2565%	0.1897%	0.3092%
Streptococcus	0.1019%	0.0000%	1.5671%	0.0000%
Klebsiella	0.0949%	0.0000%	0.0176%	0.0070%
Parasutterella	0.0914%	1.2157%	1.9992%	0.2987%
Haemophilus	0.0808%	0.0000%	0.0949%	0.0000%
Flavonifractor	0.0703%	0.5376%	0.0070%	0.6887%
Clostridium XIVb	0.0668%	0.0492%	0.1616%	0.0000%
Eggerthella	0.0597%	0.0351%	0.0141%	0.0035%
Allisonella	0.0422%	0.0000%		
Odoribacter	0.0211%	0.0035%		
Clostridium IV	0.0176%	0.0808%	0.0351%	0.0211%
Veillonella	0.0176%	0.0070%	0.0351%	0.0035%
Clostridium sensu stricto	0.0105%	0.0000%	0.1792%	0.0000%
Akkermansia	0.0105%	11.3629%	0.1019%	0.0105%
Coprococcus	0.0070%	0.0000%	0.9838%	0.0000%
Olsenella	0.0035%	0.0773%		
Butyricimonas	0.0035%	0.0632%	0.2424%	0.0000%
Anaerofustis	0.0035%	0.0000%		
Gordonibacter			0.0211%	0.0000%
Anaerotruncus			0.0141%	0.0984%
Clostridium XVIII			0.0211%	0.0000%
Turicibacter			0.0914%	0.0000%

[a] Bacterial genus not detected in the HMA mouse microbiotas are shown in red/blue. Both 32 of the 47 bacterial genera in donor 1 group and 28 of the 40 bacterial genera in donor 2 group were successfully transplanted to the recipient HMA mice.

Table S3. The human-derived microbiotas without a particular species indeed lacked the corresponding FISH staining signals.

Target Bacteria	FISH probe	Abundance (%) by Metagenomic Sequencing		FISH Signal (Yes or No)	
		Donor 1	Donor 2	Donor 1	Donor 2
Collinsella aerofaciens	Caero1378	0	0.0984	No	Yes
Clostridium sp. KLE 1755	CKLE1755	2.99743	0	Yes	No
Lachnospiraceae bacterium 1-4-56FAA	L56FAA	0	0.26028	No	Yes
Lachnospiraceae bacterium 3-1-57FAA-CT1	LCT1	0	2.85454	No	Yes
Holdemania sp. AP2	HAP2163	0.29126	0	Yes	No
Bacteroides massiliensis	Bmass989	0	2.14066	No	Yes
Bacteroides nordii	Bnord148	0.21685	0	Yes	No
Parabacteroides goldsteinii	Pgold579	0	0.31935	No	Yes
Parabacteroides merdae	Pmerd416	0	1.62258	No	Yes

[a] Representative data from at least three independent FISH experiments are shown.

Table S4. The FISH probes used in this study.

Target Bacteria		Probe	Probe Sequence (5′–3′)	Hybridization temp (°C)	Formamide concn (%, v/v)	Source
Species	Family of Bacteria					
Bacteroides caccae	Bacteroidaceae	Bcac	TAA AAC CCA TGC GGG AAA TAT ATG C	50	20	(2)
Bacteroides massiliensis	Bacteroidaceae	Bmass989	TAG CGT TTC CGC CAT ATT CGG TT	46	20	This study
Bacteroides nordii	Bacteroidaceae	Bnord148	ATC CCA TGC GGA AAT ATT ATA CCA TCG G	46	20	This study
Bacteroides ovatus	Bacteroidaceae	Bovat 1028	CCT TCA CAA CAG CCT TAC G	37	20	(3)
Bacteroides stercoris	Bacteroidaceae	Bster171	TAA TAA TCA TCC CAT GCG GGA AAA C	46	20	This study
Bacteroides thetaiotaomicron	Bacteroidaceae	Bthe198	ATAACCGAAATTCTTTAATAATAAG	35	20	This study
Bacteroides vulgatus	Bacteroidaceae	Bvulg1017	AGA TGC CTT GCG GCT TAC GGC	37	20	(3)
Bacteroides xylanisolvens	Bacteroidaceae	Bxyla686	ATCAGTGTCAGTTGCAGTCTAGT	46	55	This study
Parabacteroides goldsteinii	Porphyromonadaceae	Pgold579	ACC TCA AAT ATA CTC AAG TCA ACC AGT T	46	20	This study
Parabacteroides merdae	Porphyromonadaceae	Pmerd416	ACG TCC TCC ACT TTA TTC CCC TAT AAA A	46	20	This study
Clostridium citroniae	Clostridiaceae	Ccitr947	AGG TCA CTT TAC TGA CCG GTC AGG G	46	20	This study
Clostridium hathewayi	Clostridiaceae	Chath1100	CTC CCC AGA GTG CCC GAC TCT ACT C	46	20	This study
Clostridium symbiosum	Clostridiaceae	Csymb627	CCG ACA CTC CAG TTA AAC AGT TTC C	46	20	This study
Megamonas funiformis	Veillonellaceae	Mfuni1247	CCA CCT TAG ACG GTC GGT ACC ATA GGC C	46	20	This study
Megamonas hypermegale	Veillonellaceae	Mhype1256	GTT CCG CTC TGC CTC GCG ACT TCG C	46	20	This study
Megamonas rupellensis	Veillonellaceae	Mrupe35	CGC GTT ACT CAC CCG TTC GCG CAC T	46	55	This study

Continued on the next page.

Target Bacteria		Probe	Probe Sequence (5′–3′)	Hybridization temp (°C)	Formamide concn (%, v/v)	Source
Species	Family of Bacteria					
Adlercreutzia equolifaciens	Coriobacteriaceae	Aequo1433	GTT ACG ACT TCA CCC CCC TTA CCC TCC A	46	55	This study
Collinsella aerofaciens	Coriobacteriaceae	Caero1378	TCT CGG TTG GGC CGG CGA CTT CGG GTG C	46	20	This study
Clostridium asparagiforme	Clostridiaceae	Caspa29	AAA ACT TCA TCT AAA ATG CTT CGT T	46	20	This study
Clostridium nexile	Clostridiaceae	Cnexi59	TGT TAC GAC TTC ACC CCA GTT ATC GGT C	46	45	This study
Clostridium sp. KLE 1755	Clostridaiaceae	CKLE1755_C	CCA ATG ACG TGC CCG TAA CGG GGC ATT C	35	20	This study
Clostridiales bacterium 1-7-47FAA	Clostridiales	C47FAA	TCC CTG TCC CGA AGG AAG GGA CAC GTT A	50	20	This study
Anaerostipes hadrus	Lachnospiraceae	E.had579	GAC TTG CCA TAC CAC CTA CG	46	20	(4)
Blautia producta	Lachnospiraceae	ProCo1264	TTG GGA TTC GCT CAA CAT CGC TG	46	20	5
Lachnospiraceae bacterium 1-4-56FAA	Lachnospiraceae	L56FAA	GCC AGT CAG CCG GAT GTC AAG GGC A	46	20	This study
Lachnospiraceae bacterium 3-1-57FAA-CT1	Lachnospiraceae	LCT1	AGT TAT ACC GAT TCC ATC CGA AAA C	50	20	This study
Anaerotruncus colihominis	Ruminococcaceae	Acoli30	CGT CCG CCA CTA AGC TTA CAT TCA TCC A	46	20	This study
Faecalibacterium prausnitzii	Ruminococcaceae	Fprau0645	CCT CTG CAC TAC TCA AGA AAA AC	46	20	(6)
Flavonifractor plautii	Ruminococcaceae	Fplau583	AAT GCA GGC TGG AGG TTG AGC CCC CAG T	46	20	This study
Coprobacillus sp 29-1	Erysipelotrichaceae	Copro29_1	AAG TAC CGT CAC TTA TGA ATC ATT T	46	20	This study
Erysipelotrichaceae bacterium 6-1-45	Erysipelotrichaceae	Erys6_1_45_28	GGT GAT CCA TCC CCA CGT TCC CGT AGG G	46	55	This study
Holdemania sp. AP2	Erysipelotrichaceae	HAP2163	GAT GCC AGA AAC TAA CCT ATC CGG T	46	20	This study

Target	Probe	Probe Sequence (5′–3′)	Hybridization temp (°C)	Formamide concn (%, v/v)	Source
Clostridium	Clos571	AGC CCT GGG CTT TCA CTC CAG ACT T	46	20	(7)
Lachnospiraceae	Lac435	TCT TCC CTG CTG ATA GA	46	35	(8)
Most Bacteria	EUB338	GCT GCC TCC CGT AGG AGT	46	0	(9)
Control probe complementary to EUB338	NONEUB	ACT CCT ACG GGA GGC AGC	46	0	(10)

Table S5. The FISH probes failed to be confirmed of their specificities.

Bacteria		FISH Probe	Probe Sequence (5′–3′)	Hybridization temp (°C)	Formamide concn (%, v/v)	Source
Species	Family of Bacteria					
Bacteroides cellulosilyticus	Bacteroidaceae	Bcell24	ATC AAC CTA TTG CTA GGT CAT GCT G	46	20	This study
Alistipes senegalensis	Rikenellaceae	Asene168	GGGATGCCCCTCATGATGTTATG	46	20	This study
Alistipes shahii	Rikenellaceae	Ashah174	CCAGAACCGATGCCGGTCCTGATGT	46	20	This study
Subdoligranulum sp. 4-3-54A2FAA	Ruminococcaceae	S54A2FAA	TTAGAAAGGAGGTGATCCAGCCGCACCT	46	55	This study
Eubacterium cylindroides	Erysipelotrichaceae	Ecyli623	TCCAAAGCGTACGACAGTTGAGCTG	46	20	This study
Holdemania filiformis	Erysipelotrichaceae	Hfili563	TGGAATTCCATTGCCCTCTCCTGTACTT	46	20	This study
Phascolarctobacterium succinatutens	Acidaminococcaceae	Psucc72	CTTCGCAATTTTTACCGCTACACTTCAG	46	20	This study
Burkholderiales bacterium 1-1-47	Burkholderiales_noname	Burk1_47_C	ACGCGGGAGAAGCTTGCTTCTCCCG	46	20	This study
Parasutterella excrementihominis	Sutterellaceae	Pexcr61	TCGCCACTCGTCGCCGGAGAGAAGC	46	20	This study
Bilophila wadsworthia	Desulfovibrionaceae	Bwads55	TCGGCCCTACCGTAGACGGCTGCTCCTC	46	20	This study

References:

1. Sun Z, Huang S, Zhang M, Zhu Q, Haiminen N, Carrieri AP, Vázquez-Baeza Y, Parida L, Kim H-C, Knight R, Liu Y-Y. Challenges in benchmarking metagenomic profilers. Nat Methods 2021; 18: 618-626. doi: 10.1038/s41592-021-01141-3.
2. Momose Y, Park SH, Miyamoto Y, Itoh K. Design of species-specific oligonucleotide probes for the detection of *Bacteroides* and *Parabacteroides* by fluorescence *in situ* hybridization and their application to the analysis of mouse caecal *Bacteroides–Parabacteroides* microbiota. J Appl Microbiol. 2011; 111: 176-184. doi: 10.1111/j.1365-2672.2011.05039.x.
3. Rigottier-Gois L, Rochet V, Garrec N, Suau A, Doré J. Enumeration of *Bacteroides* species in human faeces by fluorescent *in situ* hybridisation combined with flow cytometry using 16S rRNA probes. Syst Appl Microbiol. 2003; 26: 110-118. doi: 10.1078/072320203322337399.
4. Schwiertz A, Le Blay G, Blaut M. Quantification of different *Eubacterium* spp. in human fecal samples with species-specific 16S rRNA-targeted oligonucleotide probes. Appl Environ Microbiol. 2000; 66: 375. doi: 10.1128/aem.66.1.375-382.2000.
5. Clavel T, Henderson G, Alpert C-A, Philippe C, Rigottier-Gois L, Doré J, Blaut M. Intestinal bacterial communities that produce active estrogen-like compounds enterodiol and enterolactone in humans. Appl Environ Microbiol. 2005; 71: 6077. doi: 10.1128/AEM.71.10.6077-6085.2005.
6. Suau A, Rochet V, Sghir A, Gramet G, Brewaeys S, Sutren M, Rigottier-Gois L, Doré J. *Fusobacterium prausnitzii* and related species represent a dominant group within the human fecal flora. Syst Appl Microbiol. 2001; 24: 139-145. doi: 10.1078/0723-2020-00015.
7. Lin L, Wu Q, Song J, Du Y, Gao J, Song Y, Wang W, Yang C. Revealing the *in vivo* growth and division patterns of mouse gut bacteria. Sci Adv. 2020; 6: eabb2531. doi: 10.1126/sciadv.abb2531.
8. Kong Y, He M, McAlister T, Seviour R, Forster R. Quantitative fluorescence *in situ* hybridization of microbial communities in the rumens of cattle fed different diets. Appl Environ Microbiol. 2010; 76, 6933-6938. doi: 10.1128/AEM.00217-10.
9. Amann RI, Binder BJ, Olson RJ, Chisholm SW, Devereux R, Stahl DA. Combination of 16S rRNA-targeted oligonucleotide probes with flow cytometry for analyzing mixed microbial populations. Appl Environ Microbiol. 1990; 56: 1919-1925. doi: 10.1128/aem.56.6.1919-1925.1990.
10. Wallner G, Amann R, Beisker W. Optimizing fluorescent *in situ* hybridization with rRNA-targeted oligonucleotide probes for flow cytometric identification of microorganisms. Cytometry. 1993; 1*4*: 136-143. doi: 10.1002/cyto.990140205.
